# Enantioseparation of *P*-Stereogenic 1-Adamantyl Arylthiophosphonates and Their Stereospecific Transformation to 1-Adamantyl Aryl-*H*-phosphinates

**DOI:** 10.3390/molecules28041584

**Published:** 2023-02-07

**Authors:** Bence Varga, Levente Buna, Daniella Vincze, Tamás Holczbauer, Béla Mátravölgyi, Elemér Fogassy, György Keglevich, Péter Bagi

**Affiliations:** 1 Department of Organic Chemistry and Technology, Budapest University of Technology and Economics, Műegyetem rkp. 3., H-1111 Budapest, Hungary; 2 Center for Structural Science, Chemical Crystallography Research Laboratory and Institute for Organic Chemistry, Research Centre for Natural Sciences, Magyar tudósok körútja 2., H-1519 Budapest, Hungary

**Keywords:** thiophosphonate, *H*-phosphinate, *P*-stereogenic, enantioseparation, organocatalysis

## Abstract

A focused library of 1-adamantyl arylthiophosphonates was prepared in racemic form. An enantioseparation method was developed for *P*-stereogenic thiophosphonates using (*S*)-1-phenylethylamine as the resolving agent. Under optimized conditions, three out of the five arylthiophosphonates were prepared in enantiopure form (*ee* > 99%). The subsequent desulfurization of optically active arylthiophosphonates gave the corresponding *H*-phosphinates without significant erosion of enantiomeric purity (*ee* = 95–98%). Hence, this reaction sequence can be considered an alternative method for the preparation of 1-adamantyl aryl-*H*-phopshinates. The absolute configuration of the (*S*)-1-adamantyl phenylphosphonothioic acid was assigned using single-crystal XRD and it allowed the confirmation that the removal of the P = S group proceeds with retention of configuration. The organocatalytic applicability of (*S*)-1-adamantyl phenylphosphonothioic acid was also evaluated as a *P*-stereogenic *Brønsted* acid.

## 1. Introduction

*P*-stereogenic compounds form an important group within organophosphorus chemistry. Remdesivir used for COVID-19 treatment and Pro-Tides are two recent examples for the application of this compound class in medicinal chemistry [1,2,3,4]. Besides this application, chiral organophosphorus compounds are used primarily as ligands in transition metal complexes or as organocatalysts [5,6,7,8,9,10]. All of these applications show the importance of synthetic method development for *P*-stereogenic compounds. Asymmetric synthesis, separation of racemic compounds, and stereoselective transformations of intermediates bearing a stable *P*-stereogenic center are considered as the most popular methods for the preparation of the *P*-chiral compound of interest in enantiomerically pure form [11,12,13,14]. For the latter approach, secondary phosphine oxides and *H*-phosphinates [15,16,17], along with the corresponding borane derivatives, are usually the intermediates of choice [18,19,20] as they have good bench and configurational stability, as well as containing one or two functional group(s) that can be displaced in a stereospecific manner.

Considering the preparation of chiral *H*-phosphinates, separation of the covalent diastereomers of menthyl *H*-phosphinates or optical resolutions using either chiral HPLC or resolving agents are the most frequently used methods [17,18,21,22,23,24,25]. As *H*-phosphinates usually lack acidic or basic functional groups, enantioseparation via diasteromeric complex formation applying neutral resolving agents is the method of choice [25]. On the other hand, phosphothionates can be regarded as acidic derivatives of *H*-phosphinates that can be separated into their enantiomers using chiral bases [26,27,28,29,30,31,32,33]. Moreover, a few studies suggested that desulfuration is feasible without significant erosion of optical activity to give *H*-phosphinates [27,34,35].

Another characteristic of phopshothionates is that they contain a stable acidic *P*-stereogenic center, and in this regard these compounds can be considered as chiral *Brønsted* acids. *P*-contatining *Brønsted* acids found wide-spread application as organocatalysts, and BINOL-based *C*_2_-symmetric derivatives are benchmark catalysts [36,37]. A few studies suggested that *C*_1_-symmetric phopshothionates can also be used as organocatalysts, but in those instances, the catalyst scaffold contained other functional groups besides the P(S)OH moiety, and it was postulated that this combined activation is responsible for the asymmetric induction [38,39,40,41]. Very recently, Montchamp et al. synthesized a series of cyclic thiophosphorus acids, and the organocatalytic activity of those compounds was tested [42].

Our recent research interest involved the preparation of optically active *P*-stereogenic >P(O)H intermediates, such as secondary phosphine oxides and *H*-phosphinates [25,43]. Our previous paper described the direct enantioseparation of chiral *H*-phosphinates (**1**), but the scope of that method was limited towards *H*-phosphinates (**1**) bearing a substituted aryl moiety [25]. Thus, the current aim of our work was to develop a complementary optical resolution strategy for such compounds (**1**) using the corresponding arylthiophosphonates (**2**) as key intermediates. In this paper, we intended to demonstrate a complete procedure incorporating the synthesis of racemic arylthiophosphonates (**2**) and their enantioseparation, as well as a desulfuration step to prepare enantiomerically pure *H*-phosphinates (**1**). We also intended to apply (*S*)-1-adamantyl phenylthiophosphonic acid [(*S*)-**2a**] as an organocatalyst in order to verify the catalytic performance of an acyclic *P*-stereogenic phopshothionate.

## 2. Results and Discussion

**Scheme 1:**
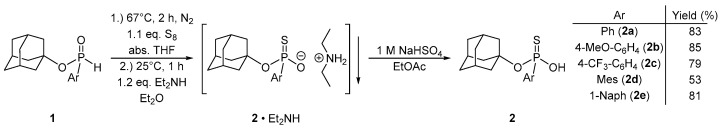
Preparation of racemic 1-adamantyl arylthiophosphonic acid derivatives (**2**).

From the methods available for the synthesis of thiophosphonic acids [44,45], we selected racemic 1-adamantyl aryl-*H*-phosphinates (**1**) as starting materials for the preparation of the corresponding arylthiophosphonic acid derivatives (**2**). The 1-adamantyl aryl-*H*-phosphinates (**1**) were synthesized according to a literature procedure [24]. In our previous paper [25], the synthesis of 1-adamantyl phenylthiophosphonic acid (**2a**) was reported by reacting 1-adamantyl phenyl-*H*-phosphinate (**1a**) with elemental sulfur at room temperature in the presence of triethylamine. However, it was found that the reaction is faster, and the purification is more efficient, when the *H*-phosphinates (**1**) are refluxed in THF with elemental sulfur without any base, and the crude thiophosphonate (**2**) is purified as a diethylamine salt (**2**ꞏEt_2_NH), which is followed by an acidic liberation step [46]. Using this procedure, racemic 1-adamantyl arylthiophosphonic acids (**2**) were obtained in yields of 53–85% (Scheme 1). The bulky alkoxy group was necessary to give sufficient configurational stability to the intermediates and the *H*-phosphinate enantiomers (**1**) throughout the complete process [24].

**Scheme 2:**
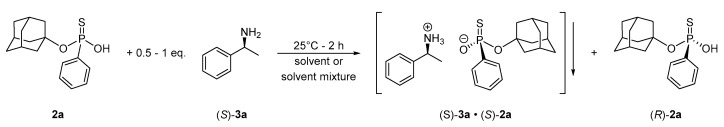
Development of an enatioseparation for 1-adamantyl arylthiophosphonates (**2**).

With the racemic materials (**2**) in hand, we continued our study by developing an enantioseparation method for the thiophosphonate derivatives (**2**) using the 1-adamantyl phenylthiophosphonic acid (**2a**) as the model compound. On the basis of related resolution procedures reported in the literature [28,29,30,31,32], (*S*)-1-phenylethylamine ((*S*)-**3a**) was selected as the resolving agent to develop an optical resolution for the thiophosphonate library (**2**). The solvent and the amount of resolving agent were two key parameters investigated initially (Scheme 2). First, diastereomers were crystallized from the corresponding solvent (EtOEt, EtOH, CH_2_Cl_2_, CHCl_3_, acetone, toluene, EtOAc), and it was found that efficient separation of thiophosphonate enantiomers ((*S*)-**2a** and (*R*)-**2a**) is not feasible when one equivalent of resolving agent ((*S*)-**3a**) is used, despite the wide variety of solvents tried, as the maximal enantiomeric excess of (*S*)-**3a** was 21%, and in the majority of instances of either racemic product was obtained or no crystallization occurred (see the Supplementary Information for details). On the other hand, the resolution became effective when the half-equivalent of (*S*)-1-phenylethylamine ((*S*)-**3a**) along with an anti-solvent were used to facilitate the crystallization of diastereomers. These results indicated that the separation of (*S*)-**2a**·(*S*)-**3a** diastereomer from (*R*)-thiophosphonate antipode ((*R*)-**2a**) (i.e., half equivalent method) can be performed more effectively than the separation of the corresponding two diastereomers ((*S*)-**2a**·(*S*)-**3a** and (*R*)-**2a**·(*S*)-**3a**) (i.e., equivalent method).

In our hands, the following procedure worked for the preparation of enantiopure (*S*)-1-adamantyl phenylthiophosphonic acid ((*S*)-**2a**): the dichloromethane solution of racemic thiophosphonate **2a** and that of (*S*)-1-phenylethylamine ((*S*)-**3a**) were mixed, which was followed by the addition of hexane anti-solvent. Crystals appeared during the agitation of the reaction mixture, and the suspension was aged for an additional 2 h, followed by the isolation diastereomer (*S*)-**2a**·(*S*)-**3a** by filtration. Intriguingly, no additional purification of the diastereomeric intermediate ((*S*)-**2a**·(*S*)-**3a**) was necessary to increase the optical purity, as pure diastereomer was obtained after a single crystallization. It is worth mentioning that ^31^P NMR could be used to determine the diastereomeric purity, as two well-resolved peaks were visible on the ^31^P spectra of the corresponding thiophosphonate (**2**) in the presence of (*S*)-1-phenylethylamine ((*S*)-**3a**). The optically active thiophosphonate **2a** was liberated from the diastereomer by acidic treatment followed by and extraction with EtOAc. After the evaporation of the solvent, the (*S*)-1-adamantyl phenylthiophosphonic acid ((*S*)-**2a**) was prepared in enantiopure form (*ee* > 99%) in a yield of 32%. It is noteworthy that crystallization did not occur when water was present, even in trace amounts. Thus, dry solvents were used in order to get consistent results. A few attempts were made to improve this resolution procedure, by changing the amount of solvent, using different halogenated solvent component, or by performing the resolution according to the equivalent method. However, none of these trials was successful (see the Supplementary Information for details).

In a separate set of experiments, the resolution with other basic resolving agents such as quinine, cinchonine, amines, or amino acid esters incorporating a chiral ethylamino moiety was also attempted according to the half equivalent method using a mixture of dichloromethane and hexane as the solvent mixture. Despite the structural similarities, as well as the identical reaction conditions, no crystalline diastereomers were formed in all but one instances, and quinine afforded thiophosphonate enantiomer (*S*)-**2a** with an *ee* of 13% and in a yield of 67%.

**Scheme 3:**
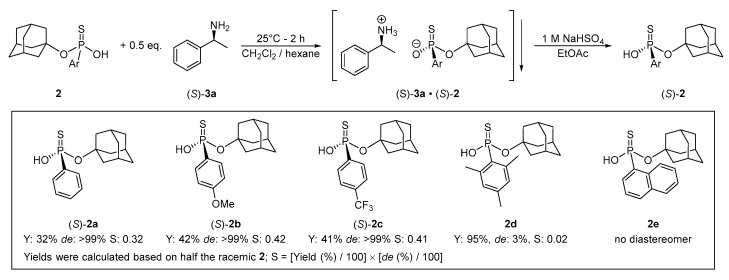
Summary of the enantioseparation results of racemic 1-adamantyl arylthiophosphonates (**2**) using (*S*)-1-phenylethylamine ((*S*)-**3a**) under optimized conditions.

After the optimization, the developed enantioseparation method using 0.5 equivalent of (*S*)-1-phenylethylamine ((*S*)-**3a**) in a mixture dichloromethane and hexane was extended to other members of the 1-adamantyl arylthiophosphonic acid library (**2b–e**). The 4-methoxy- and 4-trifluoromethyphenyl-derivatives (**2b** and **2c**) were also able to be prepared in enantiopure form (*ee* > 99%) in yields of 42% and 41%, respectively. On the other hand, the enantioseparation was unsuccessful for the mesityl and 1-naphthyl derivatives (**2d** and **2e**) as either racemic compound was obtained or no crystalline diastereomer were formed (Scheme 3).

**Figure 1:**
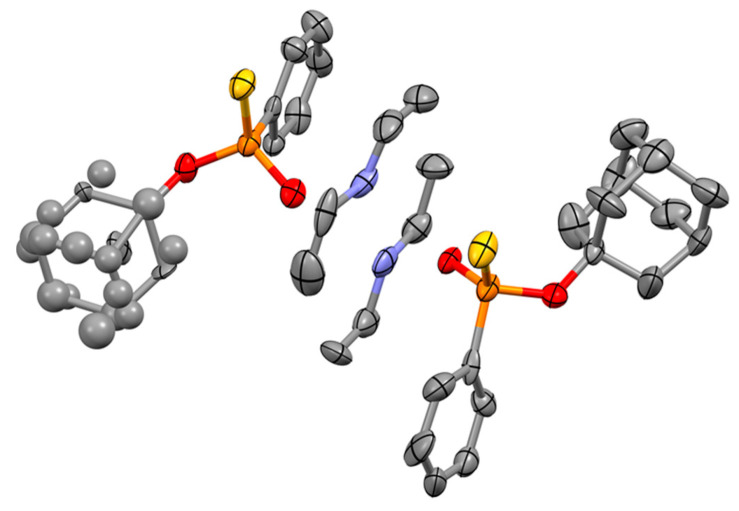
The crystallographically independent molecules in the asymmetric unit of the crystal without the atomic labelling. Hydrogens were omitted for clarity as well. Displacement ellipsoids are drawn at the 30% probability level for clarity.

Attempts were made to determine the absolute configuration of (*S*)-1-adamantyl phenylphosphonothioic acid ((*S*)-**2a**) by single crystal XRD. We failed to obtain proper crystals from thiophosphonate (*S*)-**2a**, but X-ray quality crystals could be prepared from its diethylamine salt. However, the crystals were a non-centrosymmetric twin, and the (*S*) absolute configuration of the *P*-stereogenic center was established during the measurement (Figure 1). In this manner, the (−) sign of optical rotation could be linked to an (*S*) absolute *P*-configuration for (*S*)-1-adamantyl phenylphosphonothioic acid ((*S*)-**2a**), whose correlation was postulated in our previous paper [25] on the basis of the literature analogy [21]. The absolute configurations of the (*S*)-1-adamantyl (4-methoxyphenyl)phosphonothioic acid ((*S*)-**2b**) and (*S*)-1-adamantyl (4-trifluoromethyphenyl)phosphonothioic acid ((*S*)-**2c**) could not be verified by XRD measurements, but an (*S*) absolute configuration was assigned to the (−) sign of optical rotation on the basis of structural similarity with compound (*S*)-**2a**.

**Scheme 4:**
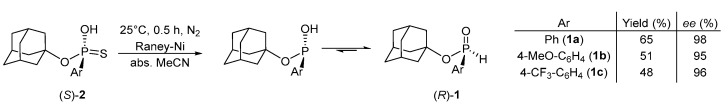
Preparation of optically active (*R*)-1-adamantyl aryl-*H*-phosphinates ((*R*)-**1a–c**) by desulfurization of enantiopure (*S*)-1-adamantyl aryphosphonothioic acids ((*S*)-**2a–c**).

The desulfurization of enantiopure (*S*)-1-adamantyl aryphosphonothioic acids ((*S*)-**2a–c**) was performed with Raney^®^-Nickel. It was found that the careful removal of the basic water from the nickel surface, the inert atmosphere, and the acetonitrile solvent were all necessary to have a clean reaction profile. When the washing steps and the use of N_2_ atmosphere were omitted, several side products were formed, probably due to hydrolysis under basic conditions and partial oxidation of *H*-phoshinates in the presence of oxygen. It was also observed during optimization that the desulfuration may take a few hours when *H*-phosphinates ((*R*)-**1**) were stirred with Raney^®^-Nickel, and upon this period, the enantiomeric purity of the corresponding *H*-phosphinates ((*R*)-**1**) dropped significantly. However, when the desulfurization reactions were run under ultrasound irradiation at room temperature, the reaction rate increased, full conversion was reached in 30 min, and the racemization was effectively suppressed [47,48]. The corresponding optically active *H*-phosphinates ((*R*)-**1a–c**) were prepared with *ee*-s in between 95 and 98% and in yield of 48–65% after purification by column chromatography (Scheme 4). It was confirmed that this desulfurization reaction proceeds with a retention of *P*-configuration, as the absolute configuration of *H*-phosphinate (*R*)-**1a** and thiophosphonate ((*S*)-**2a**) was determined by single-crystal XRD in our previous [25] and current paper. This stereochemical outcome aligns with research of others on >P(O)H compounds [27,47].

**Scheme 5:**
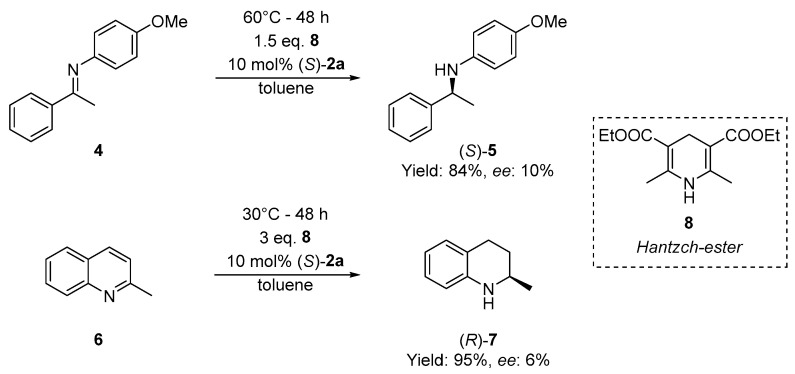
Asymmetric transfer hydrogenation of *N*-(4-methoxyphenyl)-1-phenylethanimine (**4**) and 2-methylquinoline (**6**) using (*S*)-**2a** as the organocatalyst.

As the last step of the study, the applicability of (*S*)-1-adamantyl phenylthiophosphonic acid ((*S*)-**2a**) was tested as chiral *Brønsted* acid catalyst. On the basis of other studies using *P*-stereogenic acids [39,40,42], the transfer hydrogenations of *N*-(4-methoxyphenyl)-1-phenylethanimine (**4**) and 2-methylquinoline (**6**) were selected as a test reaction, and *Hantzsch* ester (**8**) was the hydrogen source. The conversion was complete in all reactions, and products ((*S*)-**5** or (*R*)-**7**) were obtained with *ee*-s up to 10% (Scheme 5). These preliminary experiments, especially in comparison with literature [39,40,42], show that *P*-stereogenic *Brønsted* acids without additional *H*-bond donors or acceptors may not induce a high level of enantiomeric excess, despite being active in these transfer hydrogenation reactions.

## 3. Materials and Methods

### 3.1. General Methods (Instruments)

The chiral amines, diethyl amine, magnesium, NaHSO_4_, sulfur, and Raney^®^-Nickel (slurry in water) were purchased from Merck Chemicals Ltd. The 4-bromobenzotrifluordie and 2-bromomesitylene were purchased from Fluorochem Ltd. The reagents were used without further purification unless otherwise stated.

The 1-adamantyl phenyl-*H*-phosphinate (**1a**), 1-adamantyl 4-methoxyphenyl-*H*-phosphinate (**1b**), 1-adamantyl 1-napthyl-*H*-phosphinate (**1e**), dicloro-adamantyloxyphosphine [24], *N*-(4-methoxyphenyl)-1-phenylethan-1-imine (**4**) [49], and diethyl 2,6-dimethyl-1,4-dihydropyridine-3,5-dicarboxylate (**8**) [50] were synthesized as described in the literature, and their analytical data were identical to the ones reported.

The solvents were purchased from Merck Chemicals Ltd. and were used without further purification. Solvents were dried according to the standard procedures [51]. Dry solvents were stored over 3 Å or 4 Å molecular sieves.

The ^31^P, ^19^F, ^13^C, and ^1^H NMR spectra were taken on a Bruker AV-300 or DRX-500 spectrometer operating at 121.5, 282.4, 75.5, and 300 or 202.5, 470.6, 125.8, and 500 MHz, respectively. The chemical shifts (δ) are given in parts per million (ppm). The chemical shifts (δ) for ^1^H and ^13^C in CDCl_3_ were referenced to 7.26 and 77.16 ppm, respectively. An 85% solution of H_3_PO_4_ was the external reference for ^31^P NMR chemical shifts. Coupling constants are expressed in Hertz (Hz). The following abbreviations are used: s = singlet, d = doublet, t = triplet, q = quadruplet, m = multiplet, dd = doublet of doublets, dq = doublet of quadruplets.

The exact mass measurements were performed using an Agilent 6230C TOF LC–MS System with an Agilent Jet Stream source in positive ESI mode (buffer: ammonium formate in water/acetonitrile; drying gas: 325 °C; capillary: 3000 V; fragmentor 100 V).

LC–MS measurements were performed using an Agilent 1100 and Agilent 6130 LC–MS system in positive and negative electrospray mode.

Melting points were obtained on a melting point apparatus and are uncorrected.

The syntheses involving air-sensitive reagents, intermediates, or products were carried out according to the *Schlenk*-techniques, under a nitrogen atmosphere in *Schlenk*-type reaction vessels [52].

Thin-layer chromatography (TLC) was performed on Merck pre-coated Silica gel 60 F_254_ aluminum plates with realization by UV irradiation.

Column chromatography was performed on Silica gel 60 with a particle size of 0.063–0.200 mm supplied by Merck. Flash column chromatography was performed using a Combi-Flash^®^ (Teledyne ISCO, Lincoln, NE, USA).

The enantiomeric excess (*ee*) values of compound **1a** were determined by chiral HPLC on a PerkinElmer Series 200 instrument using normal phase mode equipped with Phenomenex Lux (Torrance, USA) ^®^ 5μm Amylose-2 (250 × 4.6 mm), using a 50:50 mixture of hexane and ethanol as an eluent with a flow rate of 0.8 mL/min (T = 20 °C, UV detector α = 254 nm). Retention times: *t*_R1_ 7.6 min (*S*)-(−)**-1a**, *t*_R2_ 13.8 min (*R*)-(+)**-1a.**

The enantiomeric excess (*ee*) values of compound **1b** were determined by chiral HPLC on a PerkinElmer Series 200 instrument using normal phase mode equipped with Phenomenex Lux ^®^ 5 μm Cellulose-2 (250 × 4.6 mm), using a 50:50 mixture of hexane and ethanol as an eluent with a flow rate of 0.8 mL/min (T = 20 °C, UV detector α = 254 nm). Retention times: *t*_R1_ 9.0 min (*S*)-(−)**-1b**, *t*_R2_ 27.5 min (*R*)-(+)**-1b.**

The enantiomeric excess (*ee*) values of compound **1c** were determined by chiral HPLC on a PerkinElmer Series 200 instrument using normal phase mode equipped with Phenomenex Lux ^®^ 5μm Cellulose-2 (250 × 4.6 mm), using a 50:50 mixture of hexane and ethanol as an eluent with a flow rate of 0.8 mL/min (T = 20 °C, UV detector α = 254 nm). Retention times: *t*_R1_ 6.0 min (*S*)-(−)**-1c**, *t*_R2_ 7.6 min (*R*)-(+)**-1c.**

The enantiomeric excess (*ee*) values of compound **5** were determined by chiral HPLC on a PerkinElmer Series 200 instrument using normal phase mode equipped with Phenomenex Lux ^®^ 5 μm Cellulose-1 (250 × 4.6 mm), using a 95:5 mixture of hexane and ethanol as an eluent with a flow rate of 0.8 mL/min (T = 20 °C, UV detector α = 254 nm). Retention times: *t*_R1_ 9.2 min (*S*)-(−)**-5**, *t*_R2_ 9.7 min (*R*)-(+)**-5.**

The enantiomeric excess (*ee*) value of compound **7** was determined by chiral HPLC on a Thermo Fisher Scientific Finnigan Surveyor instrument using reversed-phase mode on a Phenomenex Lux ^®^ 3 μm Cellulose-3 column (250 × 4.6 mm), using a 60:40 mixture of MeCN and water (0.1% NH_4_OAc) as the eluent with a flow rate of 0.8 mL/min (T = 20 °C, UV detector α = 222 nm). Retention times: *t*_R1_ 9.3 min (*S*)-(−)**-7**, *t*_R2_ 10.7 min (*R*)-(+)**-7.**

The enantiomeric excess (*ee*) values of 1-adamantyl arylphosphonothioic acids (**2a–e**) were determined by ^31^P NMR using 20 μmol of the corresponding analyte (**2a–e**), 3.9 μL (30 μmol) (*S*)-phenylethylamine (**3a**) as CSA, and 750 μL CDCl_3_ as solvent.

Optical rotations were determined on a Perkin–Elmer 341 polarimeter.

Sonication reactions were performed in an ELMA S30 Elmasonic ultrasonic bath.

X-ray intensity data were collected on a Rigaku RAXIS-RAPID II diffractometer (using graphite monochromator; Cu-Kα radiation, λ = 0.71075 Å) in the case of crystals. Crystals of (*S*)-**2a** were measured with fiber. Crystal Clear [53] (developed by Rigaku Company) software was used for data collection and refinement. Numerical and empirical absorption corrections [54] were applied to the data. The structures were solved by direct methods. Anisotropic full-matrix least-squares refinements were performed on F^2^ for all non-hydrogen atoms. Hydrogen atoms bonded to C atoms were placed in calculated positions and refined in a riding-model approximation. The computer programs used for the structure solution, refinement, and analysis of the structures were Shelx [55,56], Sir2014 [57], Wingx [58], Platon [59], and OLEX [60].

Crystallographic data (including structure factors) for the crystal structure of (*S*)-**2a** has been deposited with the Cambridge Crystallographic Data Centre as supplementary publication number CCDC 2206676.

### 3.2. Preparation of Racemic H-Phosphinates (***1c–d***)

#### 3.2.1. Preparation of Racemic 1-Adamantyl (4-Trifluoromethyl)-*H*-phosphinate (**1c**) (Representative Procedure I.)

The 1-adamantyl *H*-phosphinates (**1c–d**) were synthesized by a modified procedure of *D. Gatineau* et al. [24].

To a solution of 2.5 g (10 mmol) of dichloro-1-adamantyloxyphosphine in 5 mL of anhydrous THF, a solution of 10 mmol of (4-trifluoromethylphenyl)magnesium bromide in 15 mL of anhydrous THF was added dropwise over 4 h at −50 °C under nitrogen atmosphere (the (4-trifluoromethylphenyl)magnesium bromide was prepared by from 1.4 mL (10 mmol) of 4-bromobenzotrifluoride and 0.27 g (11 mmol) of Mg in 15 mL of anhydrous THF). The reaction mixture was stirred for 1 h at −50 °C. Then, it was allowed to warm to 25 °C, and it was stirred overnight. A total of 10 mL of water was added at 0 °C, and the reaction was stirred for 30 min at the same temperature. The phases were separated, and the aqueous layer was extracted with DCM (3 × 30 mL). The organic layers were combined, dried (Na_2_SO_4_), and evaporated. The crude product was purified by flash column chromatography (silica gel, gradient elution, hexane to EtOAc) to give 1.6 g (47%) of 1-adamantyl (4-trifluoromethyl)-*H*-phosphinate (**1c**) as a white solid.

mp.: 97–100 °C; ^31^P NMR {^1^H} (202.5 MHz, CDCl_3_) δ 12.0; ^1^H NMR (500 MHz, CDCl_3_) δ 7.90 (dd, *J* = 13.4, 7.9, 2H), 7.82 (d, *J* = 561.2, 1H), 7.74 (dd, *J* = 8.3, 2.8, 2H), 2.23 (s, 2H), 2.13 (d, *J* = 3.0, 7H), 1.66 (t, *J* = 3.1, 7H); ^13^C NMR {^1^H} (125.8 MHz, CDCl_3_) δ 136.0 (d, *J* = 136.2), 134.4 (dd, *J* = 32.7, 2.9), 131.6 (d, *J* = 11.9), 125.6 (dq, *J* = 14.0, 3.8), 123.7 (d, *J* = 272.5), 83.8 (d, *J* = 8.4), 44.3 (d, *J* = 4.6), 35.8, 31.3; HRMS (ESI/TOF) m/z: [M + Na]^+^ Calcd for C_17_H_20_F_3_NaO_2_P 367.1051; Found 367.1047.

#### 3.2.2. Preparation of Racemic 1-Adamantyl Mesityl-*H*-phosphinate (**1d**)

The 1-adamantyl mesityl-*H*-phosphinate (**1d**) was prepared according to Representative Procedure I. described in Section 3.2.1. by reacting 2.5 g (10 mmol) of dichloro-1-adamantyloxyphosphine in 5 mL of anhydrous THF with 10 mmol of 2-mesitylmagnesium bromide in 15 mL of anhydrous THF at −50 °C (the 2-mesitylmagnesium bromide was prepared by from 1.5 mL (10 mmol) of 2-bromomesitylene and 0.27 g (11 mmol) of Mg in 15 mL of anhydrous THF). The crude product was purified by flash column chromatography (silica gel, gradient elution, hexane to EtOAc) to give 1.9 g (61%) of 1-adamantyl mesityl-*H*-phosphinate (**1d**) as a white solid.

mp.: 87–90 °C; ^31^P NMR {^1^H} (202.5 MHz, CDCl_3_) δ 13.3; ^1^H NMR (500 MHz, CDCl_3_) δ 8.17 (d, *J* = 545.6, 1H), 6.85 (d, *J* = 4.5, 2H), 2.56 (s, 6H), 2.27 (s, 3H), 2.21 (s, 3H), 2.14 (d, *J* = 3.0, 6H), 1.66 (t, *J* = 3.1, 6H); ^13^C NMR {^1^H} (125.8 MHz, CDCl_3_) δ 142.2 (d, *J* = 2.6), 141.4 (d, *J* = 11.4), 130.2 (d, *J* = 12.1), 125.1 (d, *J* = 140.0), 82.1 (d, *J* = 8.6), 44.1 (d, *J* = 4.8), 35.9, 31.2, 21.3, 21.2, 21.1; HRMS (ESI/TOF) m/z: [M + H]^+^ Calcd for C_19_H_28_O_2_P 319.1827; Found 319.1823.

### 3.3. Preparation of Racemic Phosphonothioic Acids (***2a–e***)

#### 3.3.1. Preparation of 1-Adamantyl Phenylphosphonothioic Acid (**2a**) (Representative Procedure II.)

A total of 6.0 g (22 mmol) of 1-adamantyl phenyl-*H*-phosphinate (**1a**) was dissolved in 45 mL of anhydrous THF, and then 0.77 g (24 mmol) of sulfur was added in one portion. The mixture was stirred at 67 °C for 2 h under nitrogen atmosphere, followed by the evaporation of the solvent. The residue was dissolved in 88 mL of diethyl ether, and 2.7 mL of (26 mmol) diethyl amine was added dropwise. The resulting suspension was stirred for 1 h at 25 °C, and then it was filtered and washed with diethyl ether (2 × 20 mL) to obtain 7.2 g (19 mmol) of **2a**·Et_2_NH as a white solid. The corresponding phosphonothioic acid **2a** was liberated from the salt using 50 mL of 1M NaHSO_4_ and 30 mL of EtOAc. Phases were separated, and the aqueous layer was extracted with EtOAc (3 × 30 mL). The organic layers were combined, dried (Na_2_SO_4_), and evaporated to give 5.6 g (83%) of 1-adamantyl phenylphosphonothioic acid (**2a**) as a waxy solid.

^31^P NMR {^1^H} (121.5 MHz, CDCl_3_) δ 69.2; ^1^H NMR (500 MHz, CDCl_3_) δ 7.92–7.87 (m, 2H), 7.49–7.46 (m, 1H), 7.43–7.39 (m, 2H), 7.05 (bs, 1H), 2.26–2.19 (m, 6H), 2.15 (bs, 3H), 1.65–1.58 (m, 6H);^13^C NMR {^1^H} (125.8 MHz, CDCl_3_) δ 136.8 (d, *J* = 156.9), 131.6 (d, *J* = 3.2), 130.3 (d, *J* = 11.9), 128.2 (d, *J* = 15.4), 85.2 (d, *J* = 10.4), 44.0 (d, *J* = 4.2), 35.9, 31.4; HRMS (ESI/TOF) m/z: [M + H]^+^ Calcd for C_16_H_22_O_2_PS 309.1078; Found 309.1074.

#### 3.3.2. Preparation of 1-Adamantyl (4-Methoxyphenyl)phosphonothioic Acid (**2b**)

The 1-adamantyl (4-methoxyphenyl)phosphonothioic acid (**2b**) was prepared according to Representative Procedure II. described in Section 3.3.1. by reacting 0.20 g (0.65 mmol) of 1-adamantyl (4-methoxyphenyl)-*H*-phosphinate (**1b**) with 0.023 g (0.72 mmol) of sulfur in 1.3 mL of anhydrous THF. The resulting phosphonothioic acid was treated with 81 µL (0.78 mmol) of diethylamine in 2.6 mL of diethyl ether to obtain 0.24 g (0.58 mmol) of **2b**·Et_2_NH as a white solid. The corresponding phosphonothioic acid **2b** was liberated from the salt with 1M NaHSO_4_ (1.5 mL) and EtOAc (3 × 2 mL) to give 0.19 g (85%) of 1-adamantyl (4-methoxyphenyl)phosphonothioic acid (**2b**) as a dense oil.

^31^P NMR {^1^H} (202.5 MHz, CDCl_3_) δ 69.5; ^1^H NMR (500 MHz, CDCl_3_) δ 7.86–7.80 (m, 2H), 6.92–6.89 (m, 2H), 5.18 (bs, 1H), 3.84 (s, 3H), 2.24–2.14 (m, 9H), 1.65–1.59 (m, 6H); ^13^C NMR {^1^H} (125.8 MHz, CDCl_3_) δ 162.4 (d, *J* = 3.6), 132.5 (d, *J* = 13.7), 128.4 (d, *J* = 163.1), 113.6 (d, *J* = 16.6), 85.4 (d, *J* = 10.2), 55.5, 44.0 (d, *J* = 4.2), 35.9, 31.4.; HRMS (ESI/TOF) *m*/*z*: [M + H]^+^ Calcd for C_17_H_24_O_3_PS 339.1184; Found 339.1181.

#### 3.3.3. Preparation of 1-Adamantyl (4-Trifluoromethylphenyl)phosphonothioic Acid (**2c**)

The 1-adamantyl (4-trifluoromethylphenyl)phosphonothioic acid (**2c**) was prepared according to Representative Procedure II. described in Section 3.3.1. by reacting 0.20 g (0.58 mmol) of 1-adamantyl (4-trifluoromethylphenyl)-*H*-phosphinate (**1c**) with 0.020 g (0.64 mmol) of sulfur in 1.2 mL of anhydrous THF. The resulting phosphonothioic acid was treated with 72 µL (0.70 mmol) of diethylamine in 2.4 mL of diethyl ether to obtain 0.22 g (0.48 mmol) of **2c**·Et_2_NH as a white solid. The corresponding phosphonothioic acid **2c** was liberated from the salt with 1M NaHSO_4_ (1.5 mL) and EtOAc (3 × 2 mL) to give 0.17 g (79%) of 1-adamantyl (4-trifluoromethylphenyl)phosphonothioic acid (**2c**) as a yellow solid.

mp.: 58–60 °C; ^31^P NMR {^1^H} (202.5 MHz, CDCl_3_) δ 66.6; ^1^H NMR (500 MHz, CDCl_3_) δ 8.01 (dd, *J* = 14.3, 8.0, 1H), 7.67 (dd, *J* = 8.2, 3.5, 1H), 5.08 (bs, 1H), 2.27–2.17 (m, 6H), 1.63 (t, *J* = 3.0, 3H); ^13^C NMR {^1^H} (125.8 MHz, CDCl_3_) δ 140.5 (d, *J* = 157.7), 133.2 (dd, *J* = 32.6, 3.2), 130.7 (d, *J* = 12.4), 125.1 (dq, *J* = 15.5, 3.7), 123.7 (d, *J* = 272.7), 86.6 (d, *J* = 10.5), 44.0 (d, *J* = 4.2), 35.7, 31.4; HRMS (ESI/TOF) m/z: [M + Na]^+^ Calcd for C_17_H_20_F_3_NaO_2_PS 399.0771; Found 399.0768.

#### 3.3.4. Preparation of 1-Adamantyl Mesitylphosphonothioic Acid (**2d**)

The 1-adamantyl mesitylphosphonothioic acid (**2d**) was prepared according to Representative Procedure II. described in Section 3.3.1. by reacting 0.20 g (0.63 mmol) of 1-adamantyl mesityl-*H*-phosphinate (**1d**) with 0.022 g (0.69 mmol) of sulfur in 1.3 mL of anhydrous THF. The resulting phosphonothioic acid was reacted with 78 µL (0.75 mmol) of diethylamine in 2.6 mL of diethyl ether to obtain 0.15 g (0.37 mmol) of **2d**·Et_2_NH as a white solid. The corresponding phosphonothioic acid **2d** was liberated from the salt with 1M NaHSO_4_ (1.5 mL) and EtOAc (3 × 2 mL) to give 0.12 g (53%) of 1-adamantyl mesitylphosphonothioic acid (**2d**) as a waxy solid.

^31^P NMR {^1^H} (202.5 MHz, CDCl_3_) δ 63.6; ^1^H NMR (500 MHz, CDCl_3_) δ 6.86 (d, *J* = 5.1, 1H), 2.65 (s, 4H), 2.30 (bs, 4H), 2.26 (s, 2H), 2.18 (bs, 2H), 1.68–1.61 (m, 3H); ^13^C NMR {^1^H} (125.8 MHz, CDCl_3_) δ 140.9 (d, *J* = 3.2), 140.5 (d, *J* = 12.7), 130.8 (d, *J* = 156.0), 130.8 (d, *J* = 15.3), 86.5 (d, *J* = 11.3), 44.2 (d, *J* = 4.3), 35.9, 31.4, 23.9 (d, *J* = 3.9), 21.0 (d, *J* = 1.8); HRMS (ESI/TOF) m/z: [M + H]^+^ Calcd for C_19_H_28_O_2_PS 351.1548; Found 351.1540.

#### 3.3.5. Preparation of 1-Adamantyl 1-Naphtylphosphonothioic Acid (**2e**)

The 1-adamantyl 1-naphtylphosphonothioic acid (**2e**) was prepared according to Representative Procedure II. described in Section 3.3.1. by reacting 0.20 g (0.61 mmol) of 1-adamantyl 1-napthyl-*H*-phosphinate (**1e**) with 0.021 g (0.67 mmol) of sulfur in 1.3 mL of anhydrous THF. The resulting phosphonothioic acid was treated with 76 µL (0.74 mmol) of diethylamine in 2.6 mL of diethyl ether to obtain 0.22 g (0.52 mmol) of **2e**·Et_2_NH as a white solid. The corresponding acid **2e** was liberated from the salt with 1M NaHSO_4_ (1.5 mL) and EtOAc (3 × 2 mL) to give 0.18 g (81%) of 1-adamantyl 1-naphtylphosphonothioic acid (**2e**) as a waxy solid.

^31^P NMR {^1^H} (202.5 MHz, CDCl_3_) δ 68.6; ^1^H NMR (500 MHz, CDCl_3_) δ 8.71 (d, *J* = 8.6, 1H), 8.34 (dd, *J* = 19.4, 7.1, 2H), 7.97 (d, *J* = 8.2, 1H), 7.88 (d, *J* = 8.1, 1H), 7.60 (ddd, *J* = 8.6, 6.8, 1.5, 1H), 7.53 (t, *J* = 7.3, 1H), 7.46 (td, *J* = 7.7, 3.6, 1H), 2.24 (bs, 8H), 2.13 (bs, 4H), 1.59 (bs, 7H); ^13^C NMR {^1^H} (125.8 MHz, CDCl_3_) δ 134.0 (d, *J* = 11.9), 133.2 (d, *J* = 3.5), 132.9, 132.6 (d, *J* = 13.9), 131.7 (d, *J* = 10.0), 128.9 (d, *J* = 2.0), 127.3 (d, *J* = 4.9), 127.0, 126.3, 124.5 (d, *J* = 18.1), 86.5 (d, *J* = 10.8), 44.1 (d, *J* = 4.3), 35.9, 31.5; HRMS (ESI/TOF) m/z: [M + H]^+^ Calcd for C_20_H_24_O_2_PS 359.1235; Found 359.1231.

### 3.4. Resolution of 1-Adamantyl Phenylphosphonothioic Acid (***2a***) with (S)-1-Phenylethylamine ((S)-***3a***) (Representative Procedure III.)

Over the course of this research project, it was observed that even the residual water content of the solvent may influence the outcome of the resolution in a negative manner. Thus, all the solvents used for resolution were dried according to the standard procedures. Dry solvents were stored over molecular sieves 3 Å or 4 Å.

To a solution of 0.10 g (0.32 mmol) of 1-adamantyl phenylphosphonothioic acid (**2a**) in 0.65 mL of anhydrous dichloromethane, we added 21 µL (0.16 mmol) of (*S*)-1-phenylethylamine ((*S*)-**3a**) in 0.65 mL of anhydrous dichloromethane in one portion. Then, 5.1 mL of anhydrous hexane was added dropwise over 2 min. Colorless crystalline diastereomeric salt of (*S*)-**2a**·(*R*)-**3a** appeared after 1 h of stirring at 25 °C. After standing at 25 °C for 2 h, the crystals were separated by filtration and washed with 1.7 mL of hexane to give 0.022 g (32%) of the (*S*)-**2a**·(*R*)-**3a** diastereomer. The corresponding acid (*S*)-**2a** was liberated from the salt with 1 mL of 1M NaHSO_4_ and 1 mL of EtOAc. Phases were separated, and the aqueous layer was extracted with EtOAc (3 × 1 mL). The organic layers were combined, dried (Na_2_SO_4_), and evaporated to give 0.016 g (32%) of (*S*)-1-adamantyl phenylphosphonothioic acid ((*S*)-**2a**) as a waxy solid.

The enantioseparation 1-adamantyl phenylphosphonothioic acid (**2a**) with 0.5–1 equivalent (*S*)-1-phenylethylamine ((*S*)-**3a**) in different solvents or solvent mixtures was also performed according to this representative procedure. Results are summarized in Table S1 (Supplementary Material). In cases of Entries 1–8, antisolvent was not added to the reaction mixture.

The optical resolution of 1-adamantyl phenylphosphonothioic acid (**2a**) was also attempted with various chiral amines in a mixture of dichloromethane and hexane according to the representative procedure described above. The results are summarized in Table S2 (Supplementary Material).

Resolution of 1-adamantyl arylthiophosphonates (**2**) with (*S*)-1-phenylethylamine ((*S*)-**3a**) was also performed according to the representative procedure described above. The results are summarized in Scheme 3 and Table S3 (Supplementary Material).

### 3.5. Preparation of Optically Active (R)-1-Adamantyl Aryl-H-phosphinates (***1a–c***)

#### 3.5.1. Preparation of (*R*)-1-Adamantyl Phenyl-*H*-phosphinate ((*R*)-**1a**) (Representative Procedure IV.)

A total of 3.0 g of Raney^®^-Nickel (slurry in water) was washed with 3 × 5 mL anhydrous EtOH, then with 3 × 5 mL anhydrous MeCN. The Raney^®^-Nickel was then suspended in 3 mL of anhydrous MeCN, and this suspension was degassed under a nitrogen atmosphere. Then, 0.10 g (0.32 mmol) of (*S*)-1-adamantyl phenylphosphonothioic acid ((*S*)-**2a**) (*ee* > 99%) in 2 mL of anhydrous MeCN was added in one portion. The reaction was sonicated in an ultrasonic bath for 30 min under nitrogen atmosphere. The mixture was filtered through a plug of celite and washed with MeCN (2 × 10 mL) and EtOAc (3 × 50 mL). The filtrates were combined and evaporated. The crude product was purified by flash column chromatography (silica gel, gradient elution, hexane to EtOAc) to give 0.058 g (65%) of (*R*)-1-adamantyl phenyl-*H*-phosphinate ((*R*)-**1a**) with an *ee* of 98%.

[α]_D_^25^ = +43.9 (*c* = 0.7, CHCl_3_, *ee* = 98%, *R*_P_) [α]_D_^25^(lit) = +44.3 (*c* = 1.07, CHCl_3_, *ee* = 98%, *R*_P_) [24]; Chiral HPLC: Phenomenex Lux 5 μm Amylose-2 column, hexane/ethanol (50:50), *t*_R1_ 7.6 min (*S*)-(−)**-1a**, *t*_R2_ 13.8 min (*R*)-(+)**-1a**; ^31^P{^1^H} NMR (121.5 MHz, CDCl_3_) δ 14.3 (δ_lit_ 14.1) [24]; HRMS (ESI/TOF) m/z: [M + H]^+^ Calcd for C_16_H_22_O_2_P 277.1357; Found 277.1354.

#### 3.5.2. Preparation of (*R*)-1-Adamantyl (4-Methoxyphenyl)-*H*-phosphinate ((*R*)-**1b**)

The (*R*)-1-adamantyl (4-methoxyphenyl)-*H*-phosphinate ((*R*)-**1b**) was prepared according to the Representative Procedure IV. described in Section 3.5.1., by reacting 1 mL anhydrous MeCN solution of 39 mg (0.12 mmol) of (*S*)-1-adamantyl (4-methoxyphenyl)phosphonothioic acid ((*S*)-**2b**) (*ee* > 99%) with 1.2 g of Raney^®^-Nickel (slurry in water, washed with EtOH and MeCN as described in Representative Procedure IV.) suspended in 1 mL of anhydrous MeCN. The reaction mixture was filtered through a plug of celite and washed with MeCN (2 × 5 mL) and EtOAc (3 × 20 mL). The crude product obtained after the evaporation of the solvent was purified by flash column chromatography (silica gel, gradient elution, hexane to EtOAc) to give 18 mg (51%) of (*R*)-1-adamantyl (4-methoxyphenyl)-*H*-phosphinate [(*R*)-**1b**] with an *ee* of 95%.

[α]_D_^25^ = +4.7 (*c* = 0.8, CHCl_3_, *ee* = 95%, *R*_P_); Chiral HPLC: Phenomenex Lux 5 μm Cellulose-2 column, hexane/ethanol (50:50), *t*_R1_ 9.0 min (*S*)-(−)**-1b**, *t*_R2_ 27.5 min (*R*)-(+)**-1b**; ^31^P{^1^H} NMR (202.5 MHz, CDCl_3_) δ 14.1 (δ_lit_ 14.1) [25]; HRMS (ESI/TOF) m/z: [M + H]^+^ Calcd for C_17_H_24_O_3_P 307.1463; Found 307.1457.

#### 3.5.3. Preparation of (*R*)-1-Adamantyl (4-Trifluoromethylphenyl)-*H*-phosphinate ((*R*)-**1c**)

The (*R*)-1-adamantyl (4-trifluoromethyphenyl)-*H*-phosphinate ((*R*)-**1c**) was prepared according to the Representative Procedure IV. described in Section 3.5.1. by reacting 0.5 mL anhydrous MeCN solution of 20 mg (0.075 mmol) of (*S*)-1-adamantyl (4-trifluoromethylphenyl)phosphonothioic acid ((*S*)-**2c**) (*ee* > 99%) with 1.0 g of Raney^®^-Nickel (slurry in water, washed with EtOH and MeCN as described in Representative Procedure IV.) suspended in 1 mL of anhydrous MeCN. The reaction mixture was filtered through a plug of celite and washed with MeCN (2 × 5 mL) and EtOAc (3 × 20 mL). The crude product obtained after the evaporation of the solvent was purified by flash column chromatography (silica gel, gradient elution, hexane to EtOAc) to give 8.7 mg (48%) of (*R*)-1-adamantyl (4-trifluoromethylphenyl)-*H*-phosphinate ((*R*)-**1c**) with an *ee* of 96%.

[α]_D_^25^ = +14.1 (*c* = 0.4, CHCl_3_, *ee* = 96%, *R*_P_); Chiral HPLC: Phenomenex Lux 5 μm Cellulose-2 column, hexane/ethanol (50:50), *t*_R1_ 6.0 min (*S*)-(−)**-1c**, *t*_R2_ 7.6 min (*R*)-(+)**-1c**.

### 3.6. Application of (S)-***2a*** as a Chiral Organocatalyst

#### 3.6.1. Preparation of (*S*)-4-Methoxy-*N*-(1-phenylethyl)aniline ((*S*)-**5**)

Under nitrogen atmosphere, 45 mg (0.20 mmol) of *N*-(4-methoxyphenyl)-1-phenylethan-1-imine (**4**), 76 mg (0.30 mmol) of diethyl 2,6-dimethyl-1,4-dihydropyridine-3,5-dicarboxylate (**8**), and 6.2 mg (0.020 mmol) of (*S*)-1-adamantyl-phenylphosphonothioic acid ((*S*)-**2a**) were dissolved in 1.0 mL of freshly distilled anhydrous toluene and stirred for 48 h at 60 °C. The solvent was evaporated, and the crude product was purified by flash column chromatography (silica gel, DCM) to give 38 mg (84%) of (*S*)-4-methoxy-*N*-(1-phenylethyl)aniline ((*S*)-**5**) with an *ee* of 10% as a clear oil.

[α]_D_^25^ = −0.4 (*c* = 1.9, CHCl_3_, *ee* = 10%, *S*) ([α]_D_^25^(lit) = +3.9 (*c* = 0.2, CHCl_3_, *ee* = 94%, *R*) [61]; Chiral HPLC: Phenomenex Lux 3 μm Cellulose-1 column, hexane/ethanol (95:5), *t*_R1_ 9.2 min (*S*)**-5**, *t*_R2_ 9.7 min (*R*)**-5**; ^1^H NMR (500 MHz, CDCl_3_) δ 7.42–7.35 (m, 4H), 7.27 (t, *J* = 7.4, 1H), 6.74 (d, *J* = 8.7, 2H), 6.52 (d, *J* = 8.6, 2H), 4.46 (q, 6.7, 1H), 3.82 (bs, 1H), 3.74 (s, 3H), 1.54 (d, *J* = 6.1, 3H); ^13^C{^1^H} NMR (125.8 MHz, CDCl_3_) δ 152.0, 145.6, 141.7, 128.7, 126.9, 126.0, 114.9, 114.7, 55.9, 54.4, 25.2; HRMS (ESI/TOF) m/z: [M + H]^+^ Calcd for C_15_H_18_NO 228.1388; Found 228.1383.

#### 3.6.2. Preparation of (*R*)-2-Methyl-1,2,3,4-tetrahydroquinoline ((*R*)-**7**)

Under nitrogen atmosphere, 27 µL (0.20 mmol) of 2-methylquinoline (**6**), 150 mg (0.60 mmol) of diethyl 2,6-dimethyl-1,4-dihydropyridine-3,5-dicarboxylate (**8**), and 6.2 mg (0.020 mmol) of (*S*)-1-adamantyl-phenylphosphonothioic acid ((*S*)-**2a**) were dissolved in 1.0 mL of freshly distilled anhydrous toluene and stirred for 48 h at 30 °C. The solvent was evaporated, and the crude product was purified by flash column chromatography (silica gel, DCM) to give 31 mg (95%) of (*R*)-2-methyl-1,2,3,4-tetrahydroquinoline ((*R*)-**7**) with an *ee* of 6% as a clear oil.

[α]_D_^25^ = +5.1 (*c* = 1.6, CHCl_3_, *ee* = 6%, *R*) [α]_D_^25^(lit) = +79.9 (*c* = 1.0, CHCl_3_, *ee* = 94%, *R*) [62]; Reversed Phase Chiral HPLC: Phenomenex Lux ^®^ 3μm Cellulose-3 column; MeCN/water (0.1% NH_4_OAc) (60:40) *t*_R1_ 9.3 min (*S*)**-7**, *t*_R2_ 10.7 min (*R*)**-7**.

^1^H NMR (500 MHz, CDCl_3_) δ 6.99–6.95 (m, 2H), 6.61 (td, *J* = 7.4, 1.2, 1H), 6.48 (dd, *J* = 8.3, 1.2, 1H), 3.66 (bs, 1H), 3.44–3.38 (m, 1H), 2.88–2.81 (m, 1H), 2.76–2.71 (m, 1H), 1.96–1.91 (m, 1H), 1.64–1.58 (m, 1H), 1.22 (d, *J* = 6.2, 3H); ^13^C{^1^H} NMR (125.8 MHz, CDCl_3_) δ 144.9, 129.4, 126.8, 121.2, 117.1, 114.1, 47.3, 30.3, 26.7, 22.6.

## 4. Conclusions

In conclusion, a five-membered library of 1-adamantyl thiophosphonic acids (**2**) bearing different aryl moieties was prepared in racemic form. An enantioseparation method was elaborated for this class of compounds using (*S*)-1-phenylethylamine ((*S*)-**3a**) via the formation and separation of the corresponding diastereomeric salts. Under optimized conditions the phenyl, (4-methoxyphenyl), and (4-trifluoromethyphenyl) derivatives of 1-adamantyl phosphonothioic acid ((*S*)-**2a-c**) could be prepared in enantiopure form (*ee* > 99%) in yields of 32–42%. The absolute configuration of (*S*)-1-adamantyl phenylphosphonothioic acid ((*S*)-**2a**) was determined by single-crystal XRD. The enantiopure 1-adamantyl arylphosphonothioic acids ((*S*)-**2a-c**) were treated with Raney^®^-Nickel to give the corresponding optically active *H*-phosphinates ((*R*)-**1a-c**) with *ee* of 95–98% and in a yield of 48–65%. It was also confirmed that this desulfurization step proceeds with retention on the *P*-stereogenic center. This current process may be regarded as an alternative strategy for the preparation of optically active *H*-phosphinates if direct enantioseparation attempts are unsuccessful. The (*S*)-1-adamantyl phenylthiophosphonic acid ((*S*)-**2a**) was tested as a *Brønsted* acid type organocatalyst in enantioselective transfer hydrogenations. Despite the catalyst being active, the only low levels of asymmetric induction were observed as the corresponding products ((*S*)-**5** or (*R*)-**7**) were obtained with *ee*-s up to 10%.

## Data Availability

Not applicable.

## Supplementary Materials

The following supporting information can be downloaded at https://www.mdpi.com/article/10.3390/molecules28041584/s1. Supplementary Materials contain the raw data of the resolution procedures (Tables S1–S3), evaluation of SDE behavior of 1-adamantyl phenyl-*H*-phosphinate (**1a**) and 1-adamantyl phenylphosphonothioic acid (**2a**) [63,64], experimental conditions and raw data of X-ray measuements, NMR spectra, and HPLC chromatograms.
