# Supplementary material for: Enantioseparation of P-Stereogenic 1-Adamantyl Arylthiophosphonates and Their Stereospecific Transformation to 1-Adamantyl Aryl-H-phosphinates
Source: Molecules. 2023 Feb 7;28(4):1584. doi: 10.3390/molecules28041584 (PMC9966292; doi:10.3390/molecules28041584)
Supplement: Supplementary file 1 [file molecules-28-01584-s001.zip › molecules-2192589-supplementary.pdf]

Supplementary Material

Enantioseparation of *P*-stereogenic 1-adamantyl arylthiophosphonates and their stereospecific transformation to adamantyl aryl-*H*-phosphinates

Bence Varga,[a] Levente Buna,[a] Daniella Vincze,[a] Tamás Holczbauer,[b] Béla Mátravölgyi,[a] Elemér Fogassy,[a] György Keglevich,[a] Péter Bagi[a,*]

[a] *Department of Organic Chemistry and Technology, Budapest University of Technology and Economics, Műegyetem rkp. 3., H-1111 Budapest, Hungary*

[b] Center for Structural Science, Chemical Crystallography Research Laboratory and Institute for Organic Chemistry, Research Centre for Natural Sciences, Magyar tudósok körútja 2., H-1519 Budapest, Hungary

* Corresponding authors. Tel.: +36 1 4631111/5886; fax: +36 1 4633648. Email addresses: bencevarga@edu.bme.hu and bagi.peter@vbk.bme.hu

Table of Contents

Data of the resolution procedures S2
Evaluation of SDE behavior (Self-Disproportionation of Enantiomers) in case 1-adamantyl phenyl-*H*-phosphinate (**1a**) and 1-adamantyl phenylphosphonothioic acid (**2a**) S5
X-ray measurements S7
^{31}P, ^{1}H and ^{13}C NMR spectra of the compounds prepared S9
HPLC and NMR traces of optically active compounds (**1-2**) S27

1. Data of the resolution procedures

Table S1. Resolution of 1-adamantyl phenyl-thiophosphonate (**2a**) with 0.5-1 equivalent of (*S*)-1-phenylethylamine [(*S*)-**3a**] in different solvents or solvent mixtures.

Entry	Eq.	Solvents[a]	Yield[b] (%)	*de*[c] (%)	S[d] (-)	Abs. Config.[e]
1	1	4×Et_2O	166	8	0.14	(*S*)
2	1	4×EtOH		no diastereomer		
3	1	4×CH_2Cl_2		no diastereomer		
4	1	4×$CHCl_3$		no diastereomer		
5	1	4×acetone	94	21	0.20	(*S*)
6	1	4×toluene	77	racemic	-	-
7	1	10×EtOAc	61	racemic	-	-
8	0.5	4×Et_2O	63	8	0.05	(*S*)
9	0.5	4×CH_2Cl_2 16×hexane	32	>99	0.32	(*S*)
10[f]	0.5	2×CH_2Cl_2 8×hexane	58	27	0.16	(*S*)
11	0.5	4×$CHCl_3$ 16×hexane		no diastereomer		
12	1	4×CH_2Cl_2 16×hexane	114	racemic	-	-

[a]Mixture of solvents for the crystallization and recrystallizations [mL of solvent / mmol of P(S)OH].
[b]The yield of the diastereomer was calculated based on the half of the racemic 1-adamantyl phenyl-thiophosphonate (**2a**) that is regarded to be 100% for each antipode.
[c]Determined by ^{31}P NMR.
[d]Resolving capability, also known as the Fogassy parameter [S (-) = (Yield [%] /100)×(*de* [%]/100)] [1].
[e]Absolute configuration of the 1-adamantyl phenyl-thiophosphinate (**2a**) was determined by X-ray analysis.
[f]The diastereomeric salt was purified by two recrystallization.

Table S2. Resolution of 1-adamantyl phenyl-thiophosphonate (**2a**) with various amines (**3**) in dichloromethane – hexane solvent mixture.

Entry	Resolving agent	Eq.	Solvents[a]	Yield[b] (%)	*de*[c] (%)	S[d] (-)	Abs. Config.[e]
1[f]	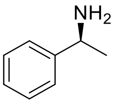	0.5	$4\times CH_2Cl_2$<br>$16\times$hexane	32	>99	0.32	(*S*)
2	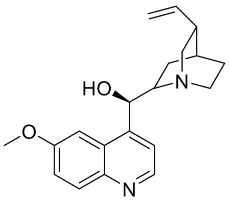	0.5	$4\times CH_2Cl_2$<br>$16\times$hexane	67	13	0.09	(*S*)
3	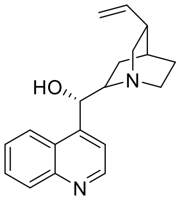	0.5	$4\times CH_2Cl_2$<br>$16\times$hexane		no diastereomer		
4	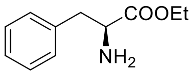	0.5	$4\times CH_2Cl_2$<br>$16\times$hexane		no diastereomer		
5	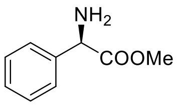	0.5	$4\times CH_2Cl_2$<br>$16\times$hexane		no diastereomer		
6	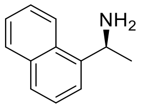	0.5	$4\times CH_2Cl_2$<br>$16\times$hexane		no diastereomer		
7	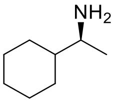	0.5	$4\times CH_2Cl_2$<br>$16\times$hexane		no diastereomer		
8	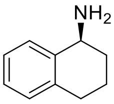	0.5	$4\times CH_2Cl_2$<br>$16\times$hexane		no diastereomer		
9	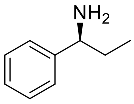	0.5	$4\times CH_2Cl_2$<br>$16\times$hexane		no diastereomer		
10	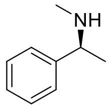	0.5	$4\times CH_2Cl_2$<br>$16\times$hexane		no diastereomer		
11	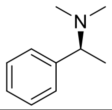	0.5	$4\times CH_2Cl_2$<br>$16\times$hexane		no diastereomer		

[a-e]See Table S1.

[f] Table S1, Entry 8.

Table S3. Resolution of 1-adamantyl arylthiophosphonates (**2**) with (*S*)-1-phenylethylamine [(*S*)-**3a**] in dichloromethane – hexane solvent mixture.

Entry	Ar	Eq.	Solvents[a]	Yield[b] (%)	*de*[c] (%)	S[d] (-)	Abs. Config.[e]
1[f]	Ph (**2a**)	0.5	4×CH_2Cl_2 16×hexane	32	>99	0.32	(*S*)
2	4-MeO-C_6H_4 (**2b**)	0.5	4×CH_2Cl_2 16×hexane	42	>99	0.42	(*S*)
3	4-CF_3-C_6H_4 (**2c**)	0.5	4×CH_2Cl_2 16×hexane	41	>99	0.41	(*S*)
4	Mes (**2d**)	0.5	4×CH_2Cl_2 16×hexane	95	3	0.02	-
5	1-Naph (**2e**)	0.5	4×CH_2Cl_2 16×hexane		no diastereomer		

[a-d]See Table S1.

[e]Absolute configuration of thiophosphinate **2a** was determined by X-ray analysis. Absolute configuration of thiophosphinates **2b-c** were assigned by structural analogy.

[f] Table S1, Entry 8.

(*S*)-1-adamantyl phenylphosphonothioic acid [(*S*)-**2a**]: $[\alpha]_D^{25}$ = −5.6 (*c* = 1.1, $CHCl_3$, *ee* = >99%, S_P).

(*S*)-1-adamantyl (4-methoxyphenyl)phosphonothioic acid [(*S*)-**2b**]: $[\alpha]_D^{25}$ = −5.1 (*c* = 2.0, $CHCl_3$, *ee* = >99%, S_P).

(*S*)-1-adamantyl (4-trifluoromethyphenyl)phosphonothioic acid [(*S*)-**2c**]: $[\alpha]_D^{25}$ = −11.0 (*c* = 1.0, $CHCl_3$, *ee* = >99%, S_P).

2. Evaluation of SDE behavior (Self-Disproportionation of Enantiomers) in case 1-adamantyl phenyl-*H*-phosphinate (1a) and 1-adamantyl phenylphosphonothioic acid (2a)

In accordance of the literature [2], the following evaluations were made to test the SDE properties of 1-adamantyl phenyl-*H*-phosphinate (**1a**) and 1-adamantyl arylphosphonothioic acid (**2a-c**), and thus the accuracy of the reported *ee* values.

During the resolution of 1-adamantyl arylphosphonothioic acids (**2a-c**), ^{31}P NMR was used to determine the diastereomereic purity of the (*S*)-**3a** · (*S*)-**2a-c** diastereomers, as two well resolved peaks were visible on the ^{31}P spectra of the corresponding thiophosphonate (**2a-c**) in the presence of (*S*)-1-phenylethylamine [(*S*)-**3a**]. After the enantiomerically pure (*S*)-**2a-c** thiophosphonates were liberated by extraction from the corresponding diasteromers, their enantiomeric purity was re-evaluated by ^{31}P NMR using *c.a.* 6 mg (20 μmol) of thiophosphonate [(*S*)-**2a-c**] as analyte, 3.9 μL (30 μmol) of (*S*)-1-phenylethylamine [(*S*)-**3a**] as chiral solvating agent and 750 μL $CDCl_3$ as solvent. These results showed parity with the ones obtained as the diastereomeric intermediate of the resolution process.

It is noteworthy, that no chiral chromatographic method could be developed for *ee* measurements of 1-adamantyl arylphosphonothioic acids (**2**) over the course of this research.

In our previous paper, the SDE behavior of 1-adamantyl phenyl-*H*-phosphinate (**1a**) was demonstrated during crystallization [3]. In (*S*)-**2** -> (*R*)-**1** transformation there was no crystallization step. However, column chromatography was identified as a potential source for SDE phenomenon. Thus, 32 mg (0,12 mmol) of enantiomeric mixture of (*R*)-1-adamantyl phenyl-*H*-phosphinate [(*R*)-**1a**] with an ee of 65% was subjected to flash column chromatographic separation on a 4 g silica column (40-63 μm). An 8 : 2 mixture of hexane and ethyl acetate was the eluent with the flow rate of 10 mL / min in isocratic mode. Fractions of 5 mL volume were collected, and their *ee* and mass were determined. Results are summarized in Figure S1, which indicates a Δee value of 3% in between the fractions, which is in the range of the general experimental and analytical error. In summary, no significant SDE behavior was identified during the chromatographic purification of 1-adamantyl phenyl-*H*-phosphinate [(*R*)-**1a**] [(*R*)-**1a**], and the ee values reported for the 1-adamantyl aryl-*H*-phosphinates [(*R*)-**1a-c**] are not biased by SDE.

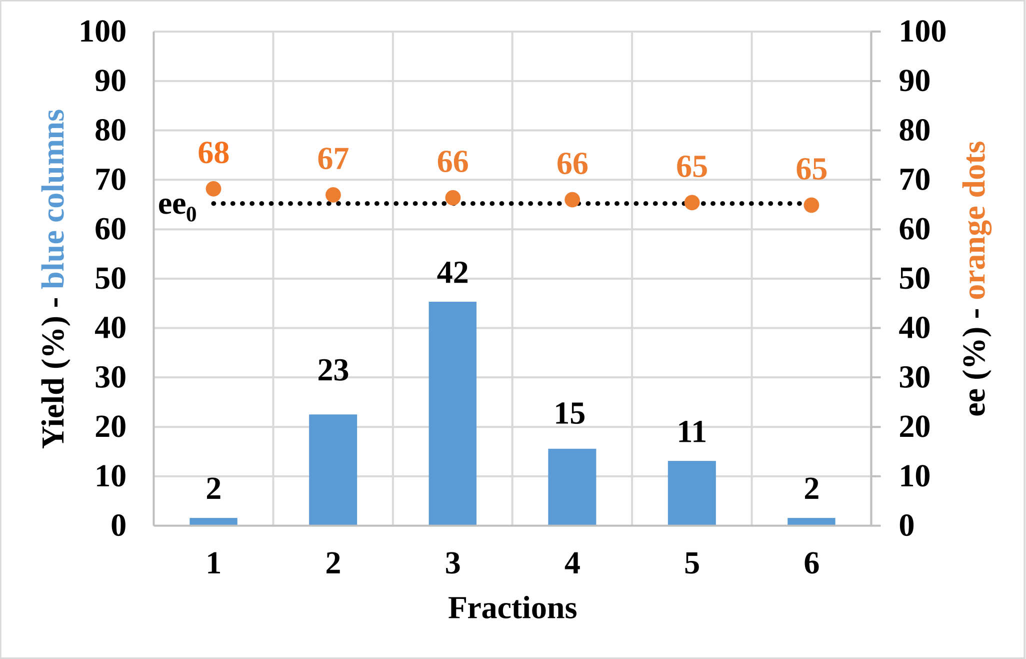

Figure S1. Yield and ee values of the fractions collected during the chromatographic purification of an enantiomeric mixture of (*R*)-1-adamantyl phenyl-*H*-phosphinate [(*R*)-**1a**] (ee_0 = 65%)

3. X-ray measurements

X-ray quality crystals were prepared in the following manner. 15 mg (0.049 mmol) of (*S*)-1-adamantyl phenylphosphonothioic acid [(*S*)-**2a**] was dissolved in 0.50 mL of diethyl ether and 5 μL (0.051 mmol) of diethylamine was added in one portion. The white precipitate formed was isolated by the removal of the solvent. 5 mg of this salt was dissolved in 0.50 mL of dichloromethane, and X-ray quality crystals were formed during the slow evaporation of the solvent.

Crystal data: $C_{40}H_{64}N_2O_4P_2S_2$, *Fwt.*: 762.99, colourless, platelet, size: 0.50 x 0.40 x 0.10 mm, tetragonal, space group $P\ 4_3$, a = 9.9248(3)Å, b = 9.9248(3)Å, c = 41.8666(12)Å, α = 90°, β = 90°, γ = 90°, V = 4123.9(3)Å^3, T = 103(2)K, Z= 4, F(000) = 1648, D_x = 1.229 Mg/m^3, μ 2.221mm^{-1}.

A crystal of (*S*)-**2a** was mounted on a glass fiber. Cell parameters were determined by least-squares using 110567 (3.165 ≤ θ ≤ 68.366°) reflections.

Intensity data were collected on a(n) Rigaku R-AXIS-RAPID II diffractometer (monochromator; Cu-*K*α radiation, λ = 1.54187Å) at 103(2) K in the range 4.224 ≤ θ ≤ 68.226.

A total of 148578 reflections were collected of which 148578 were unique [*R(*σ) = 0.2553]; intensities of 94577 reflections were greater than 2σ(*I*). Completeness to θ = 1.000.

A numerical absorption correction was applied to the data (the minimum and maximum transmission factors were 0.884092 and 0.977873).

The structure was solved by direct methods (and subsequent difference syntheses).

Anisotropic full-matrix least-squares refinement on F^2 for all non-hydrogen atoms yielded R_1 = 0.0902 and wR^2 = 0.2221 for 1332 [I>2σ(I)] and R_1 = 0.1315 and wR^2 = 0.2532 for all (148578) intensity data, (number of parameters = 462, goodness-of-fit = 1.055, the maximum and mean shift/esd is 0.009 and 0.001). The absolute structure parameter is 0.032(12). (Friedel coverage: 0.971, Friedel fraction max.: 1.000, Friedel fraction full: 1.000).

The maximum and minimum residual electron density in the final difference map was 1.14 and -0.23e.Å^{-3}.

The weighting scheme applied was $w = 1/[\sigma^2(F_o^2)+(0.11390.0000P)^2+0.0000P]$ where $P = (F_o^2+2F_c^2)/3$.

Details of crystallographic data, data collection and refinement for crystal (*S*)-**2a** is collected in Table S4.

Table S4. Summary of crystallographic data, data collections, structure determination and refinement for (*S*)-**2a**.

Structure	(*S*)-**2a**
CCDC number	2206676
Empirical formula	$C_{16}H_{20}O_2P_1S_1^-$, $C_4H_{12}N_1^+$
Formula weight	762.99
Temperature (K)	103(2)
Radiation	Cu-Kα
Crystal system	tetragonal
Space group	$P\ 4_3$
Unit cell dimensions	
a (Å)	*a* =9.9248(3)
b (Å)	*b* =9.9248(3)
c (Å)	*c* =41.8666(12)
Volume	4124(1)$Å^3$
Z, Z'	4, 2
Density (calculated)	1.229 Mg/m^3
Absorption coefficient, μ	2.221 mm^{-1}
F(000)	1648
Crystal colour, description	colourless, platelet
Crystal size	0.50 x 0.40 x 0.10 mm
Absorption correction	numerical
Max. and min. transmission	0.884092 and 0.977873
θ–range for data collection	$4.224 \leq \theta \leq 68.226°$
Index ranges	$-11 \leq h \leq 11$; $-11 \leq k \leq 11$; $-50 \leq l \leq 50$
Reflections collected	148578
Completeness to 2θ	1.000
Absolute structure parameter	0.032(12)
Friedel coverage	0.971
Friedel fraction max.	1.000
Friedel fraction full	1.000
Independent reflections	148578
Reflections $I>2\sigma(I)$	94577
Data / restraints / parameters	148578 /19 /462
Goodness-of-fit on $F2$	1.055
Final *R* indices [$I>2\sigma(I)$]	R_1 =0.0902, wR^2 =0.2221
R indices (all data)	R_1 =0.1315, wR^2 =0.2532
Max. and mean shift/esd	0.009;0.001
Largest diff. peak and hole	1.14;-0.23 e.$Å^{-3}$

^{31}P, ^{1}H and ^{13}C NMR spectra of the compounds prepared

1-adamantyl phenyl-*H*-phosphinate (**1a**)

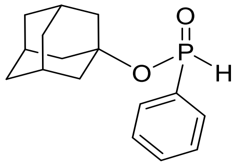

^{31}P{^{1}H} NMR (121.5 MHz, $CDCl_3$)

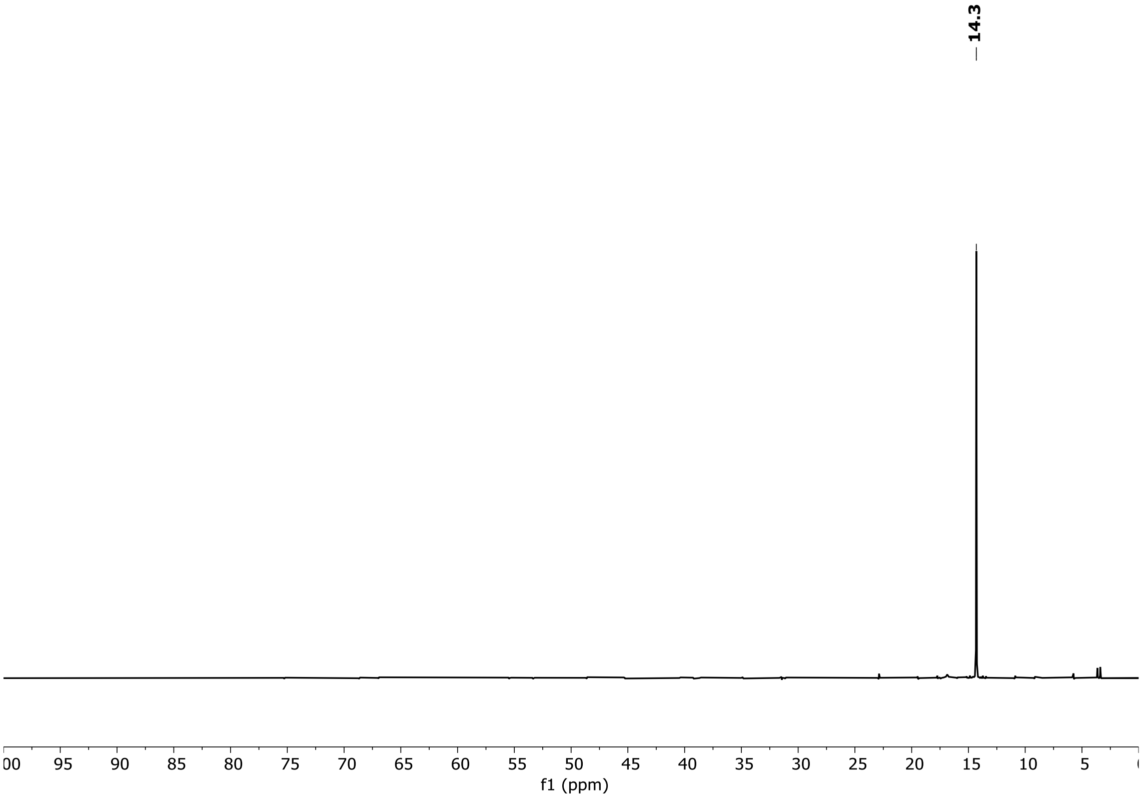

1-adamantyl (4-methoxyphenyl)-*H*-phosphinate (**1b**)

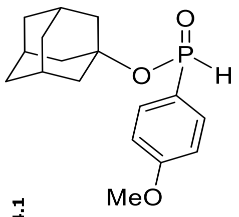

$^{31}P\{^{1}H\}$ NMR (202.5 MHz, $CDCl_3$)

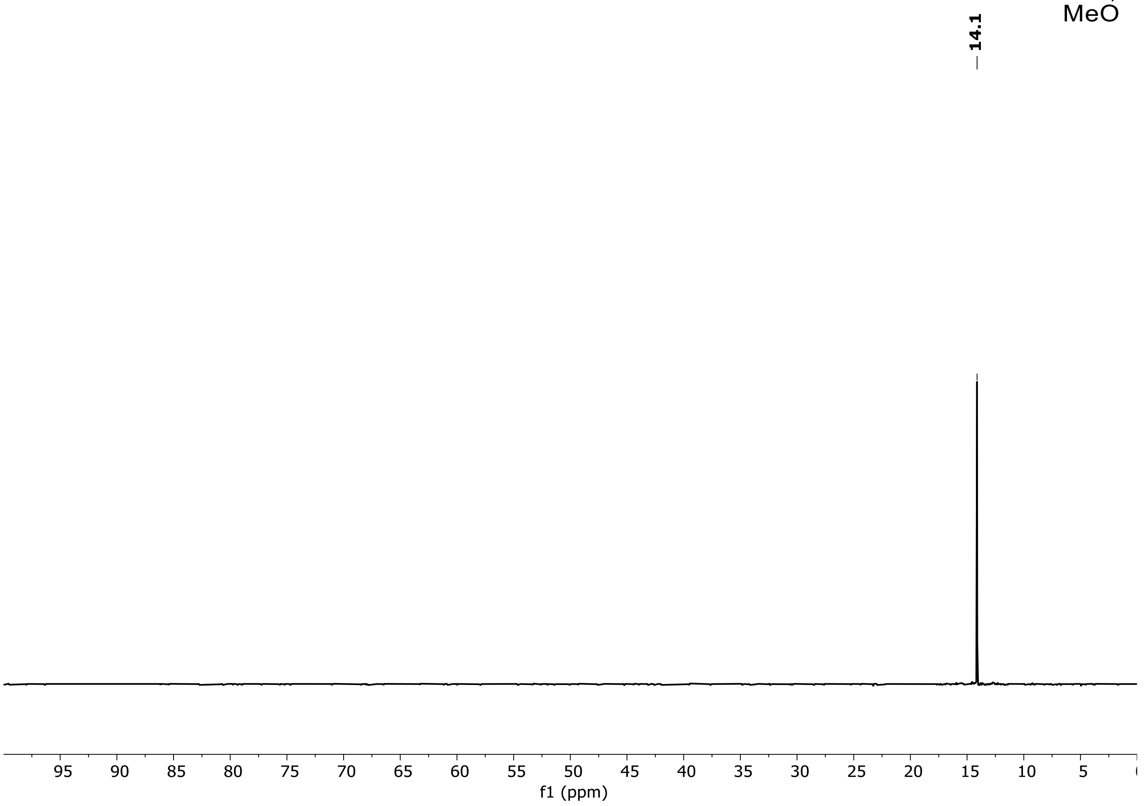

1-adamantyl (4-trifluoromethylphenyl)-*H*-phosphinate (**1c**)

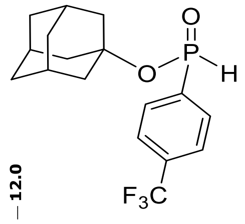

^{31}P NMR {^{1}H} (202.5 MHz, $CDCl_3$)

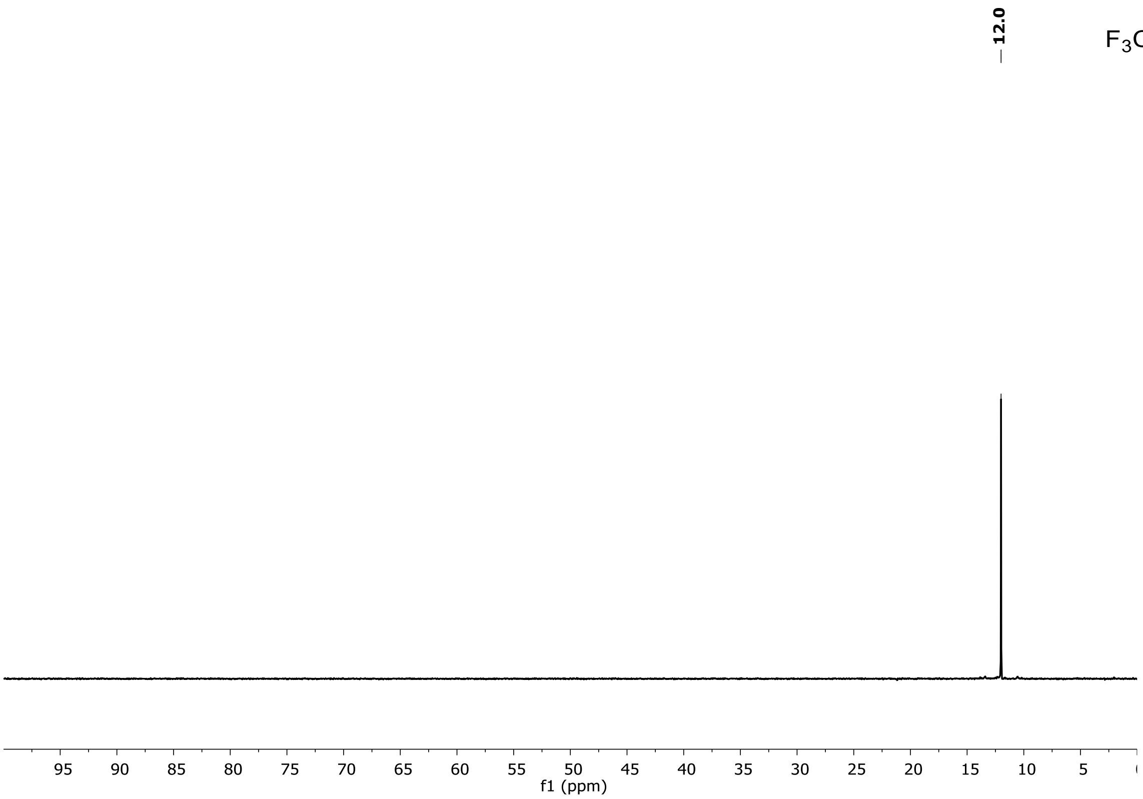

^{1}H NMR (500 MHz, $CDCl_3$)

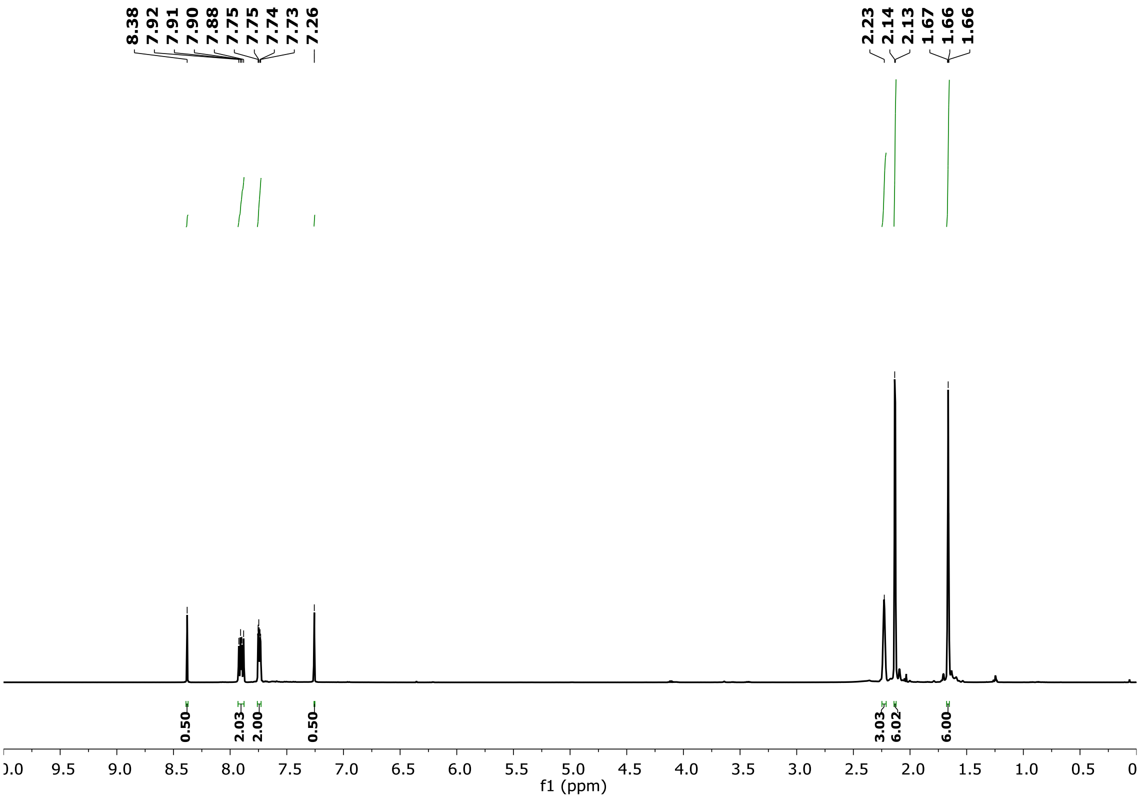

^{13}C NMR {^{1}H} (125.8 MHz, $CDCl_3$)

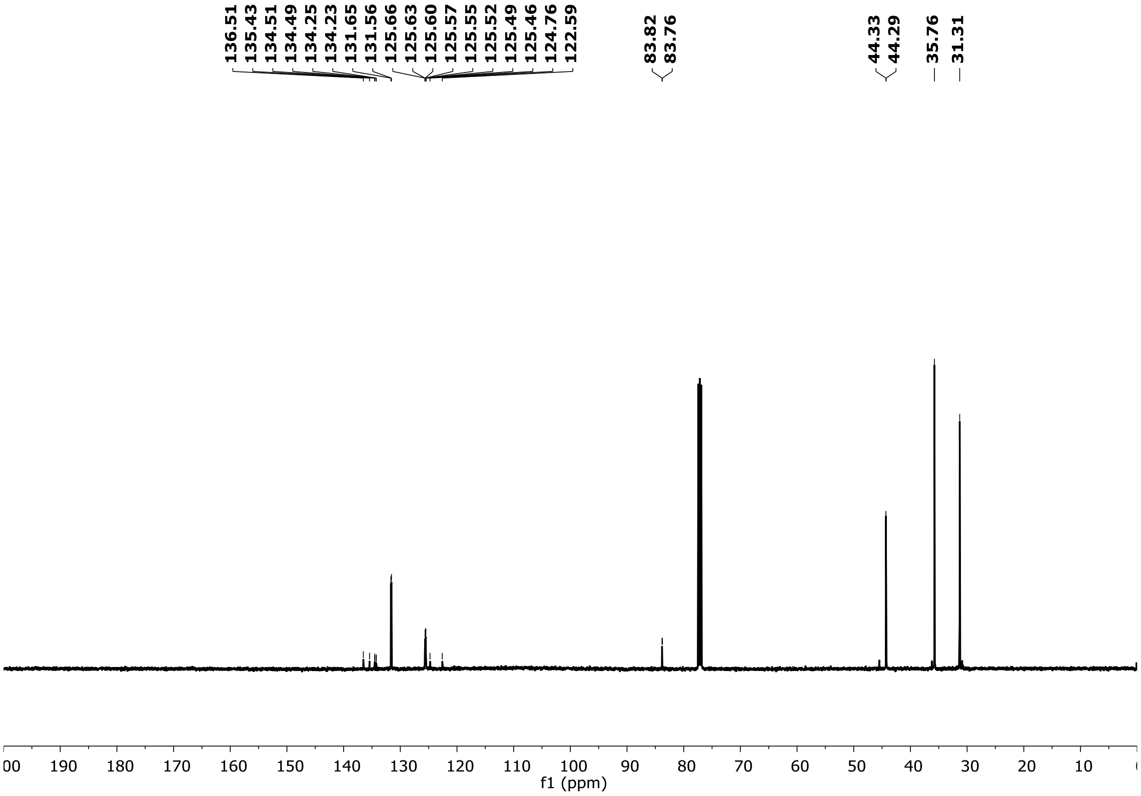

1-adamantyl mesityl-*H*-phosphinate (**1d**)

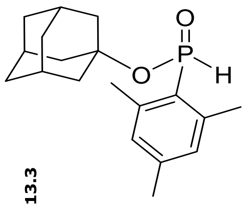

^{31}P NMR {^{1}H} (202.5 MHz, $CDCl_3$)

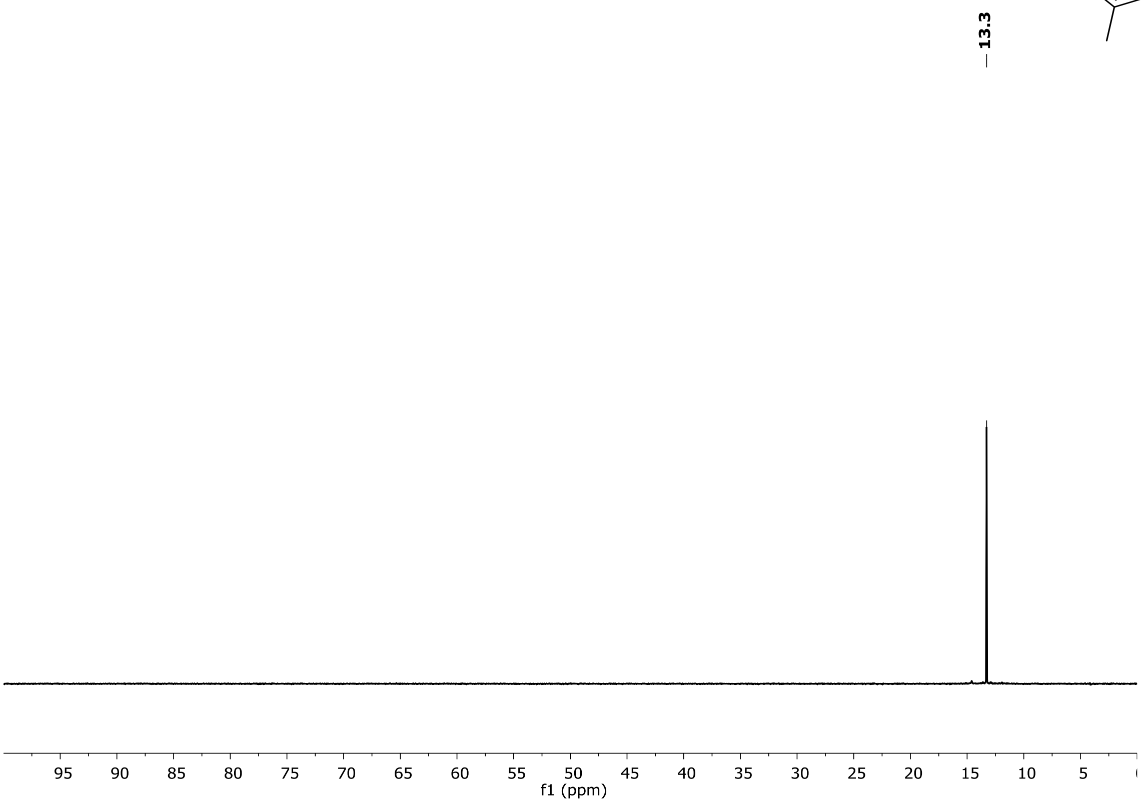

^{1}H NMR (500 MHz, $CDCl_3$)

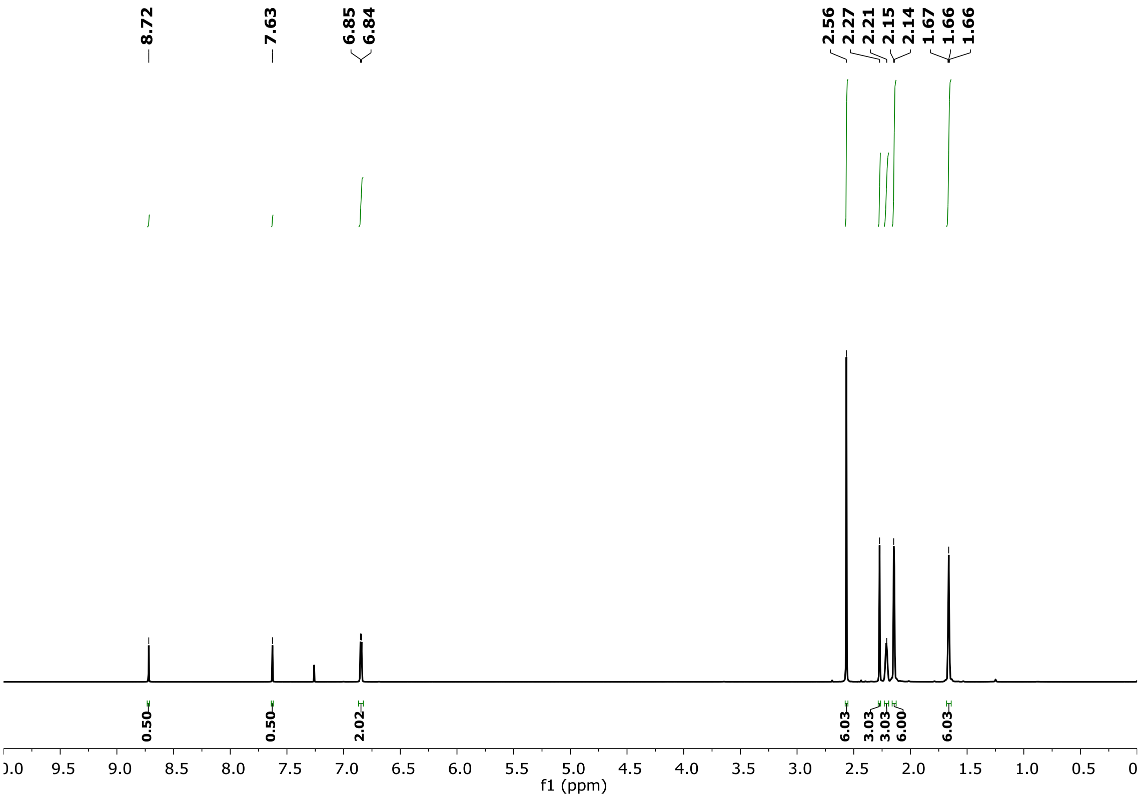

^{13}C NMR {^{1}H} (125.8 MHz, $CDCl_3$)

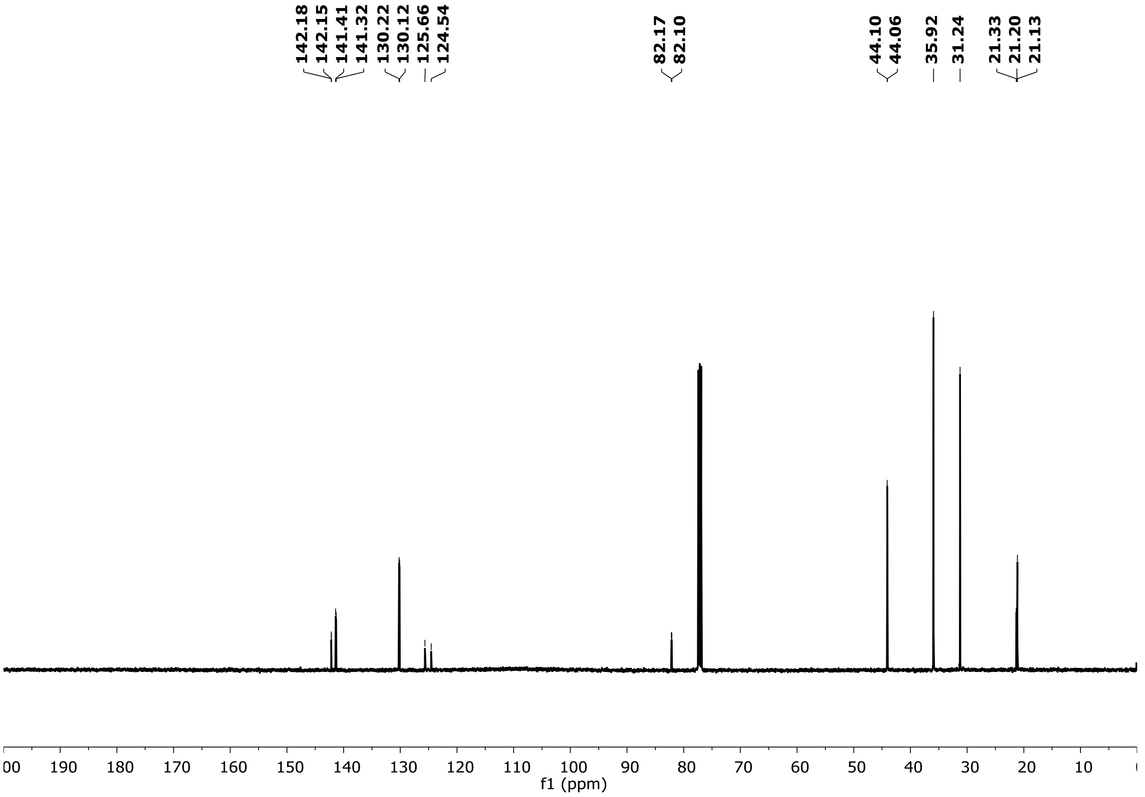

1-adamantyl phenylphosphonothioic acid (**2a**)

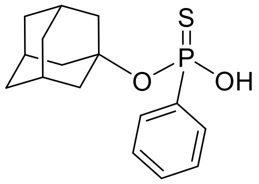

^{31}P NMR {^{1}H} (202.5 MHz, $CDCl_3$)

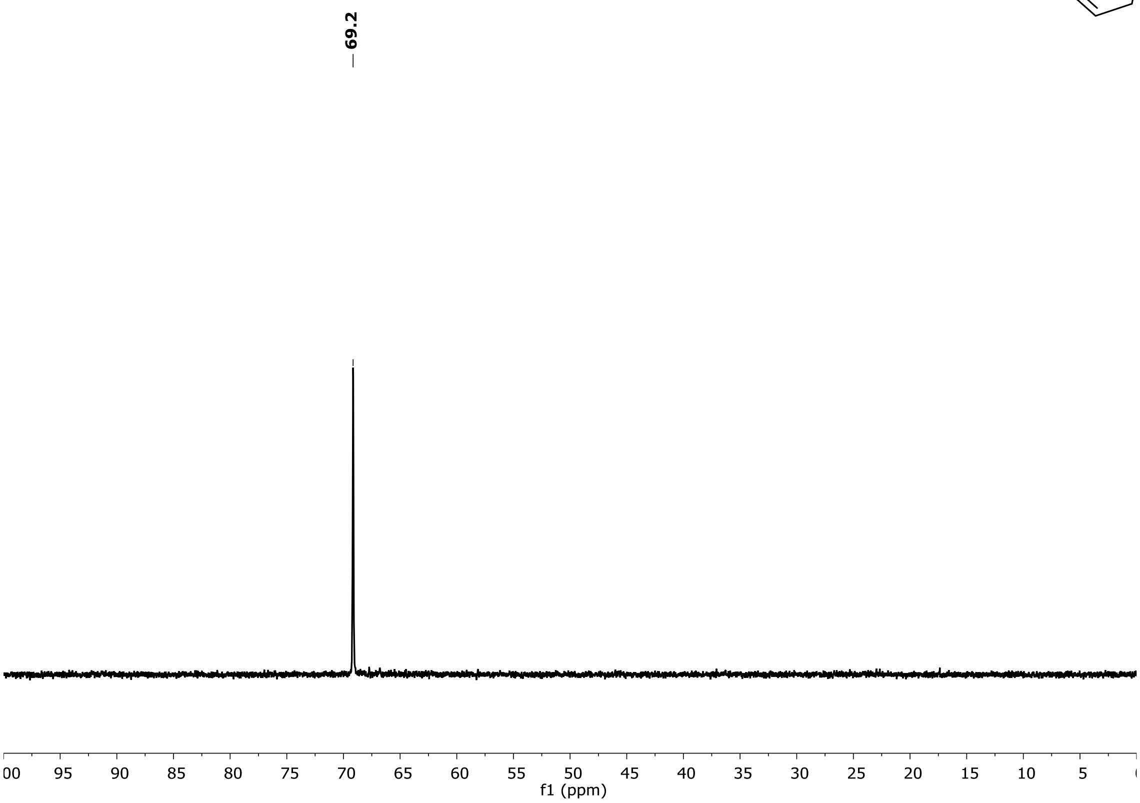

^{1}H NMR (500 MHz, $CDCl_3$)

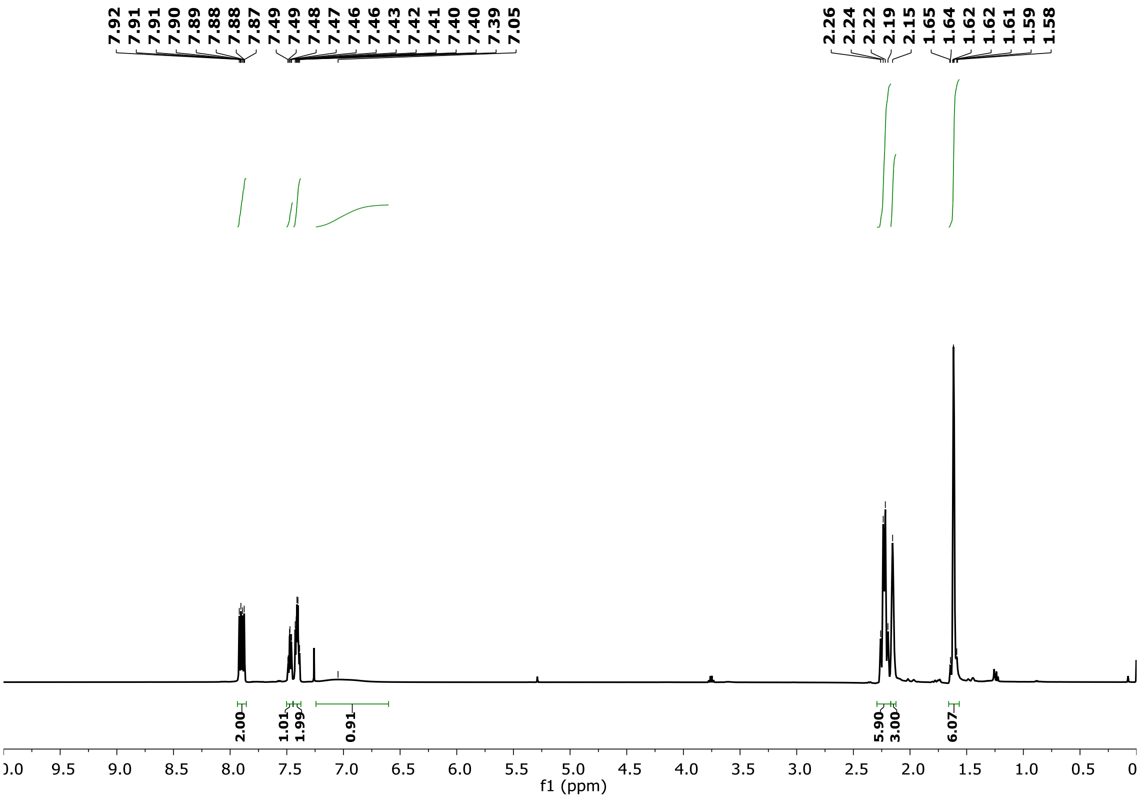

^{13}C NMR {^{1}H} (125.8 MHz, $CDCl_3$)

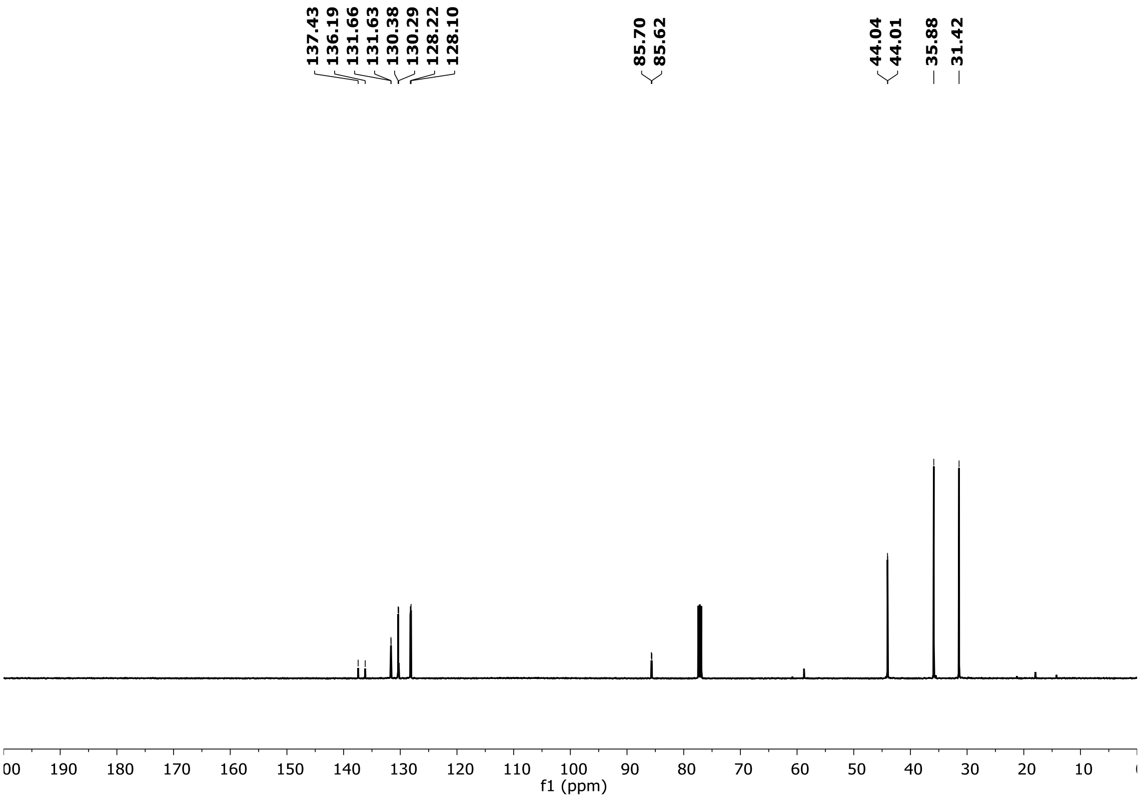

1-adamantyl (4-methoxyphenyl)phosphonothioic acid (**2b**)

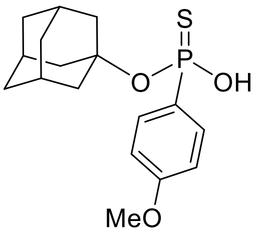

^{31}P NMR {^{1}H} (202.5 MHz, $CDCl_3$)

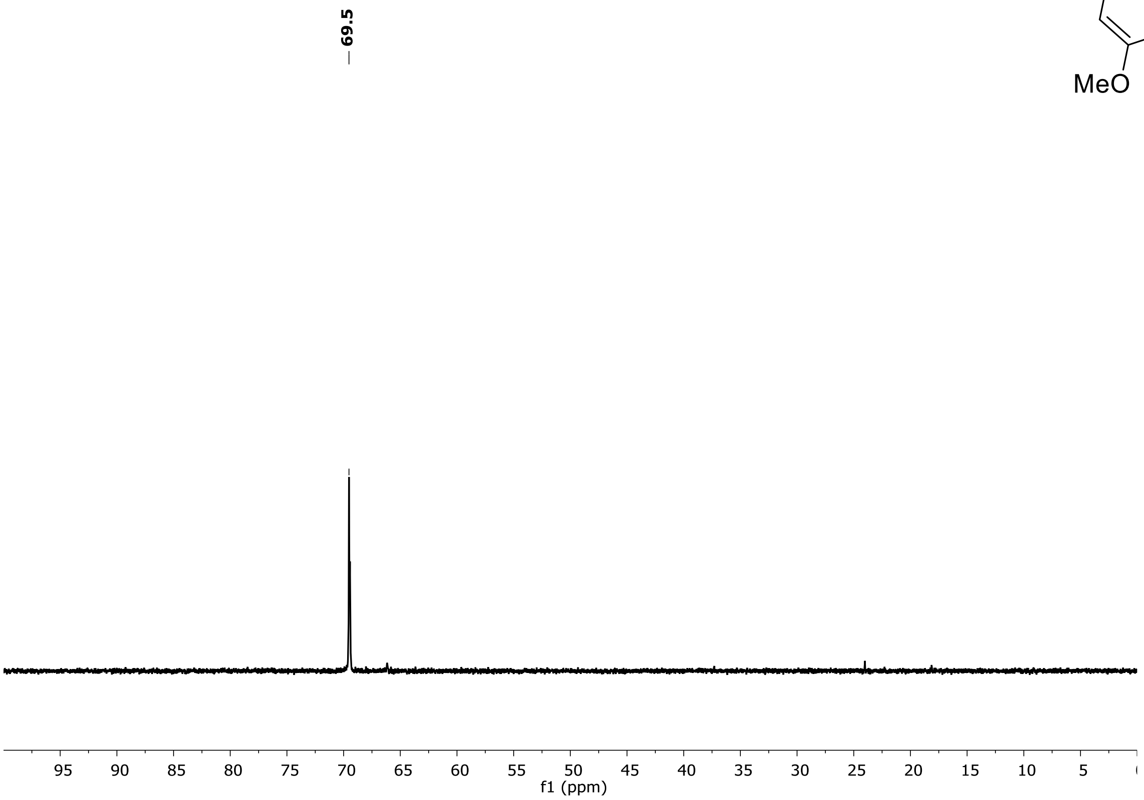

^{1}H NMR (500 MHz, $CDCl_3$)

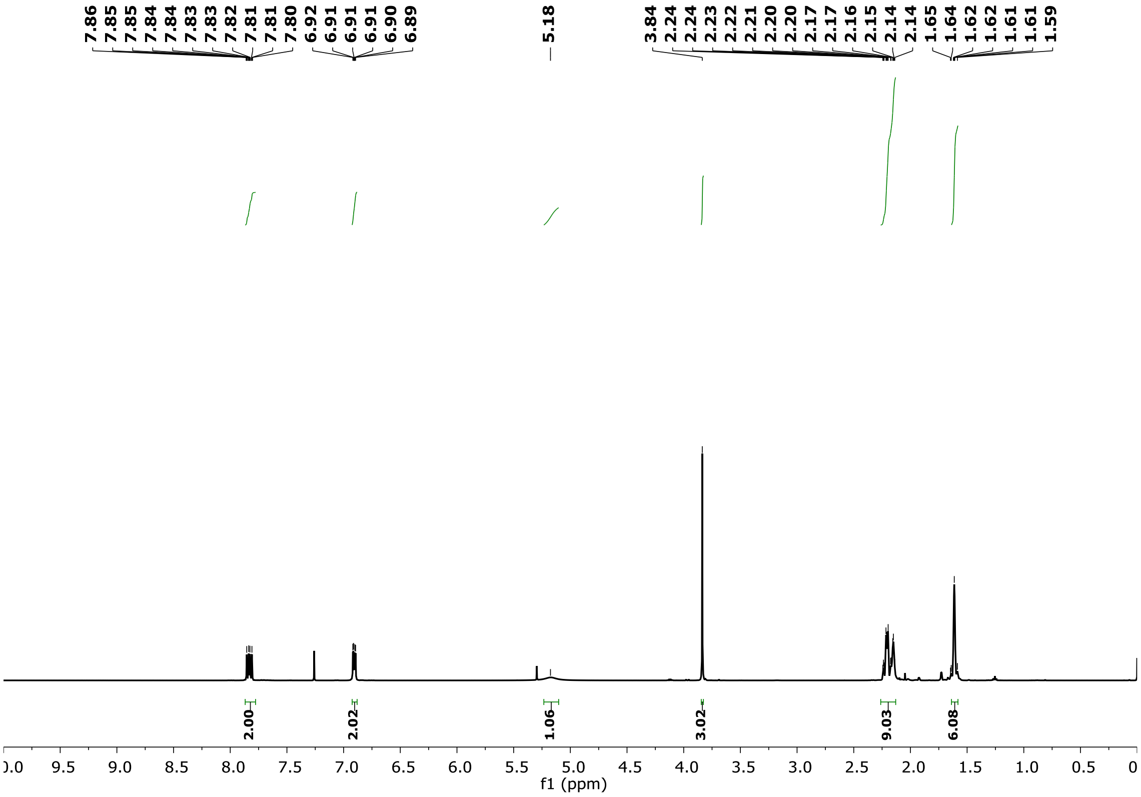

^{13}C NMR {^{1}H} (125.8 MHz, $CDCl_3$)

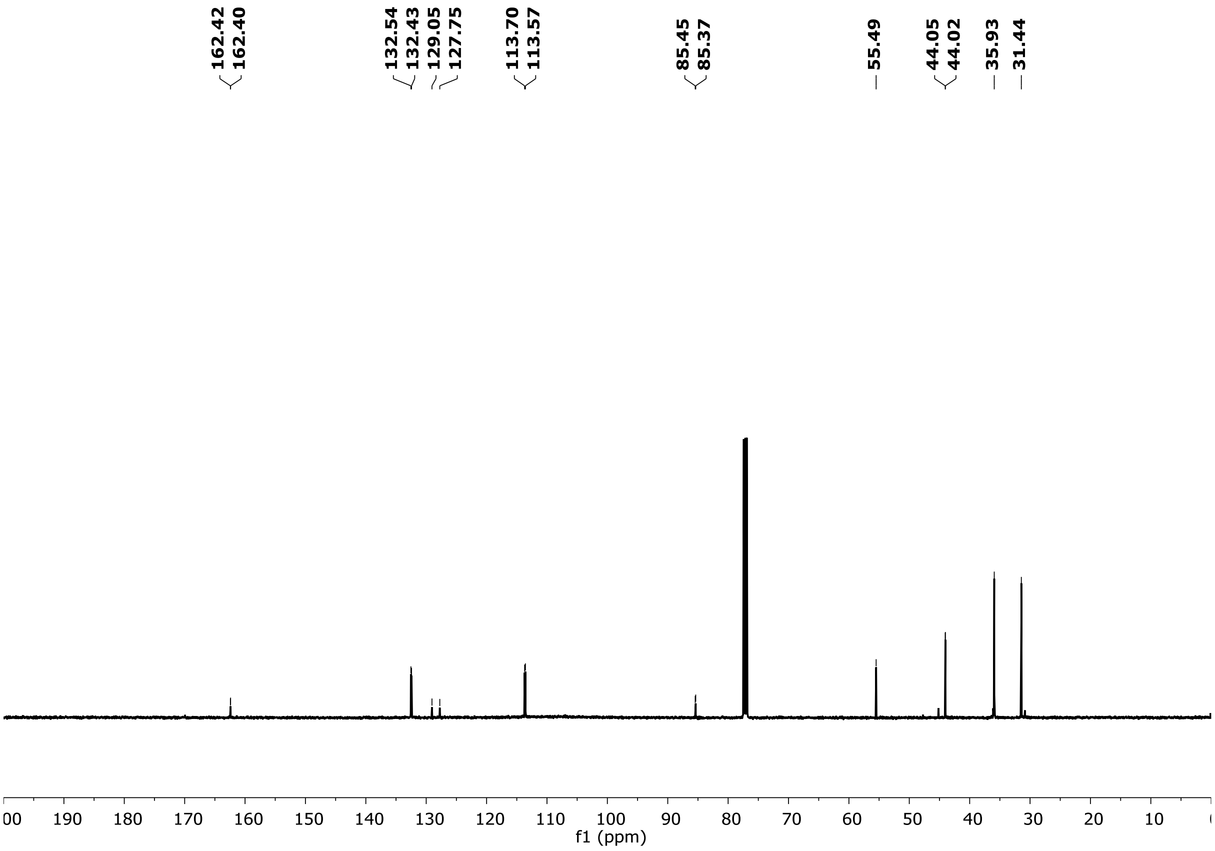

1-adamantyl (4-trifluoromethylphenyl)phosphonothioic acid (**2c**)

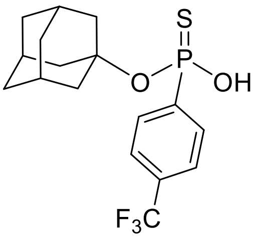

^{31}P NMR {^{1}H} (202.5 MHz, $CDCl_3$)

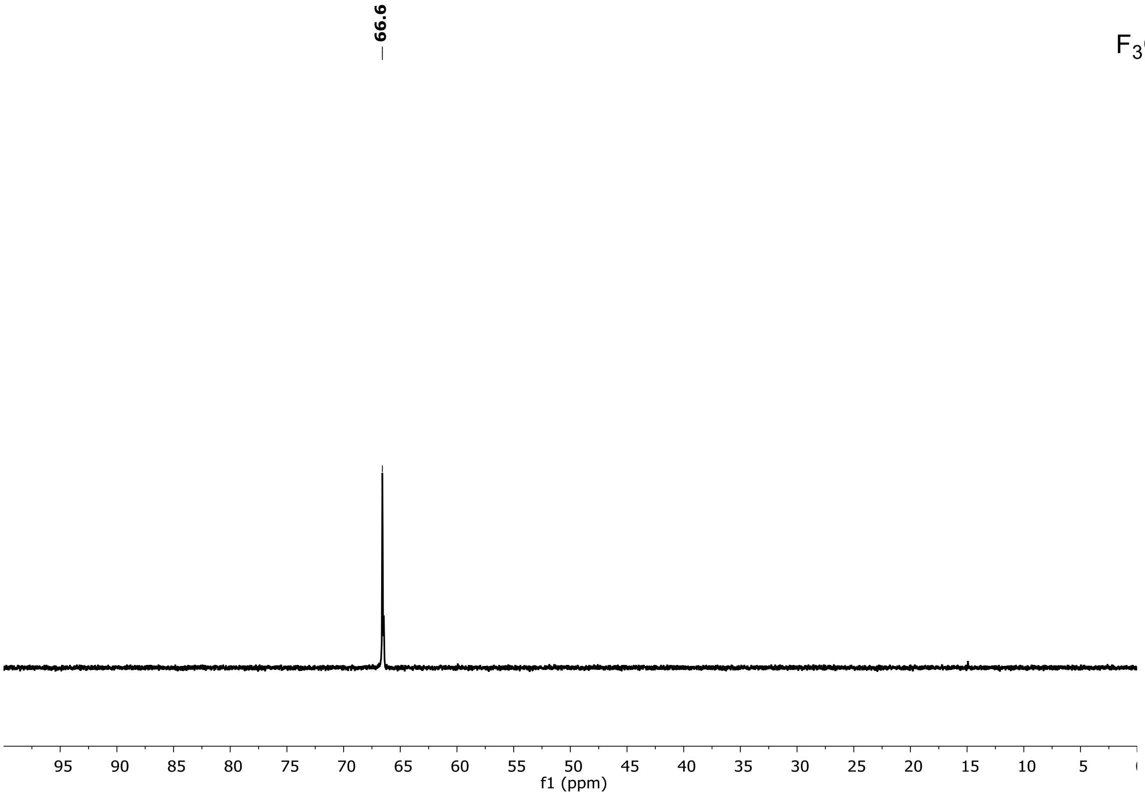

^{1}H NMR (500 MHz, $CDCl_3$)

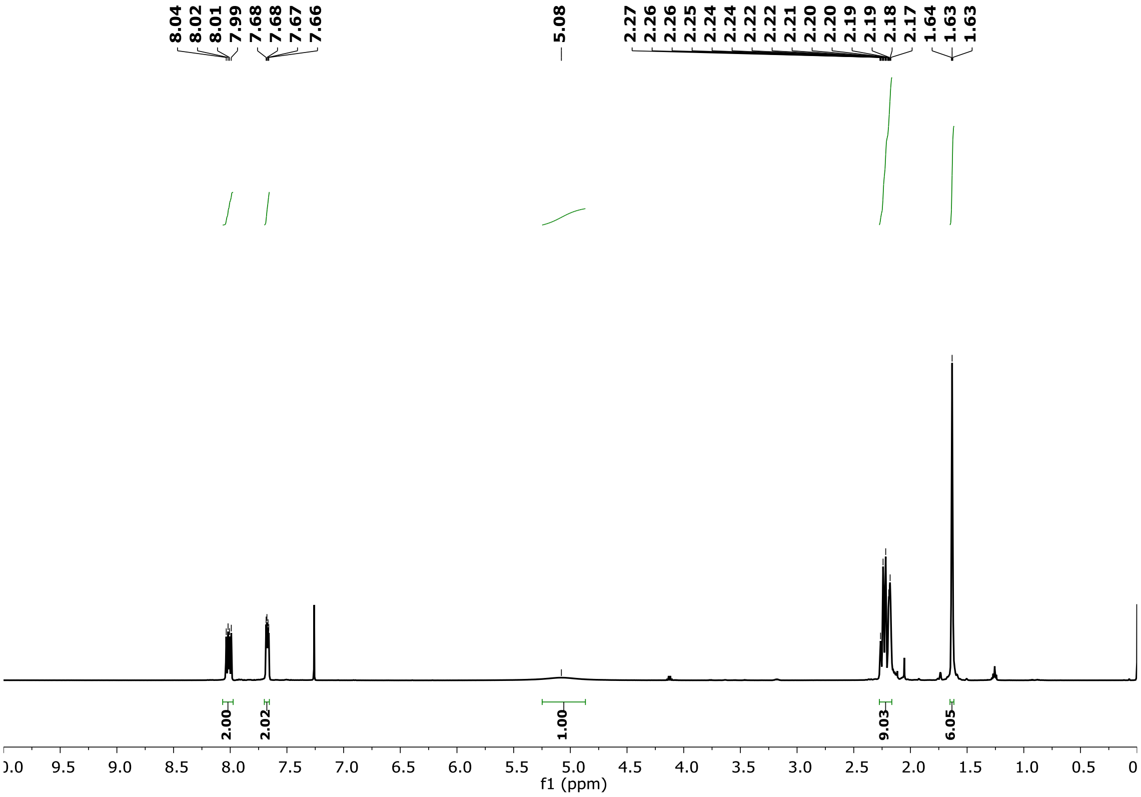

^{13}C NMR {^{1}H} (125.8 MHz, $CDCl_3$)

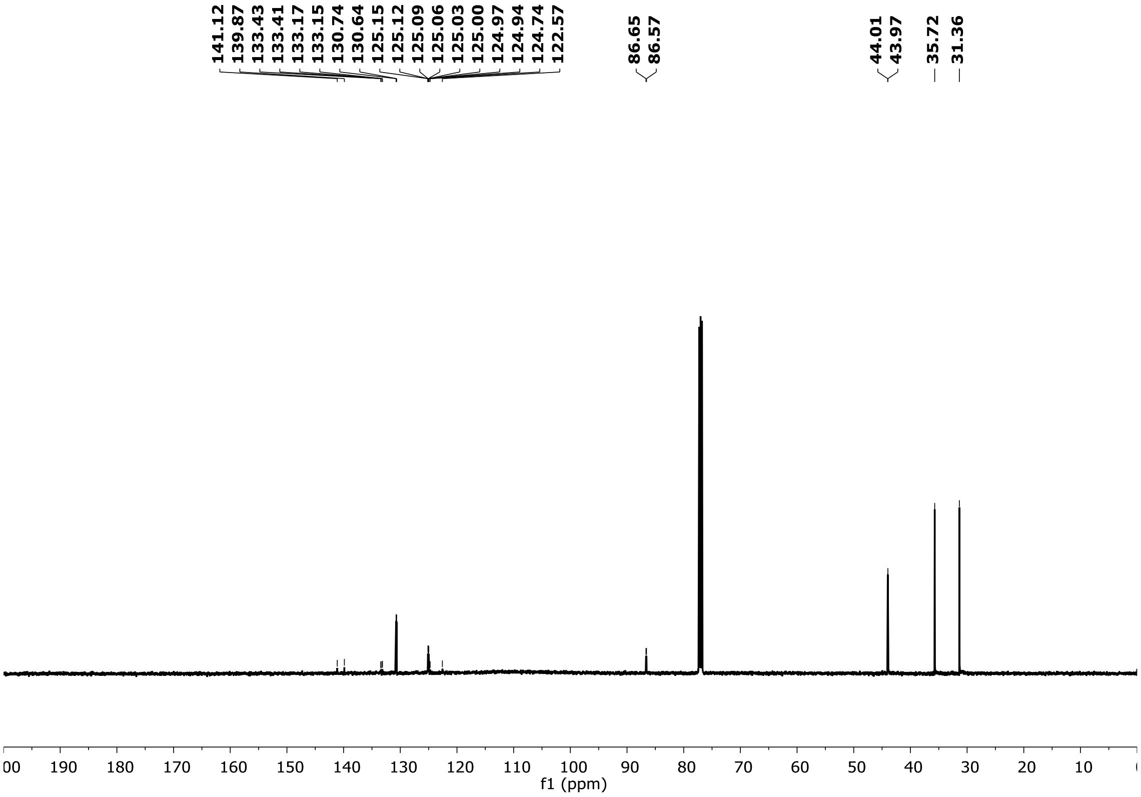

1-adamantyl mesitylphosphonothioic acid (**2d**)

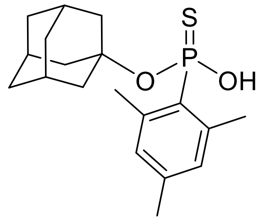

^{31}P NMR {^{1}H} (202.5 MHz, $CDCl_3$)

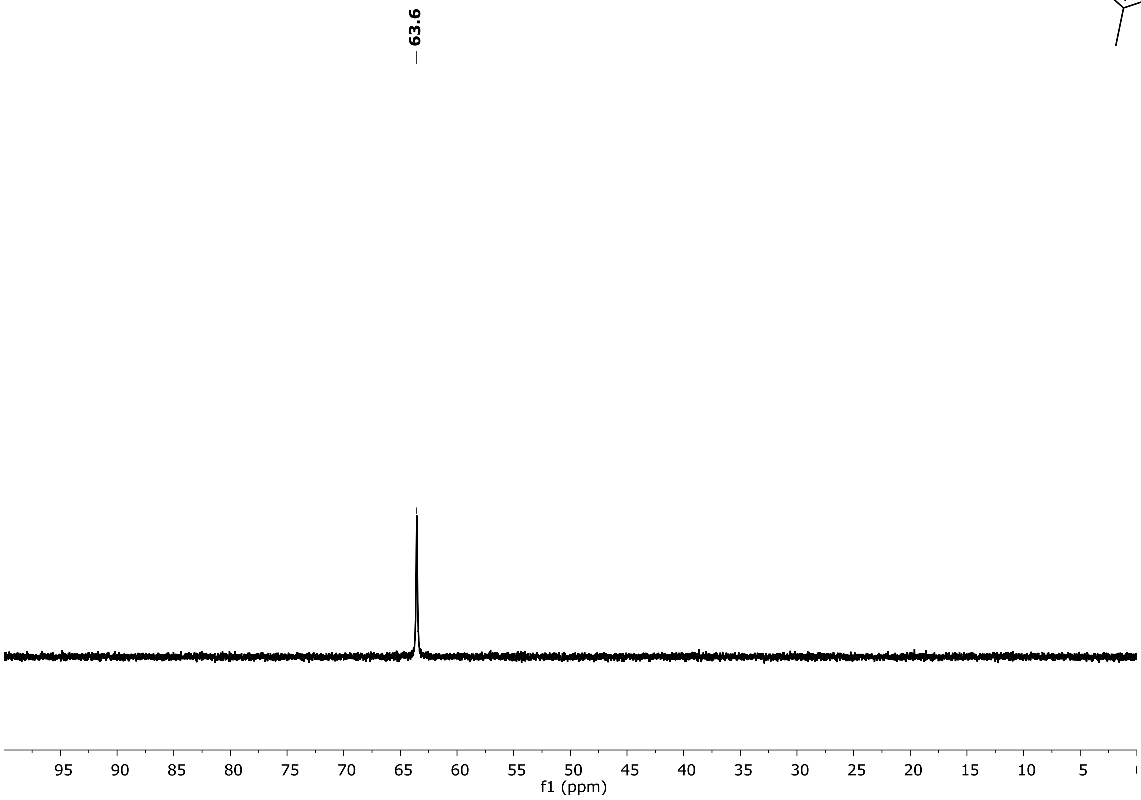

^{1}H NMR (500 MHz, $CDCl_3$)

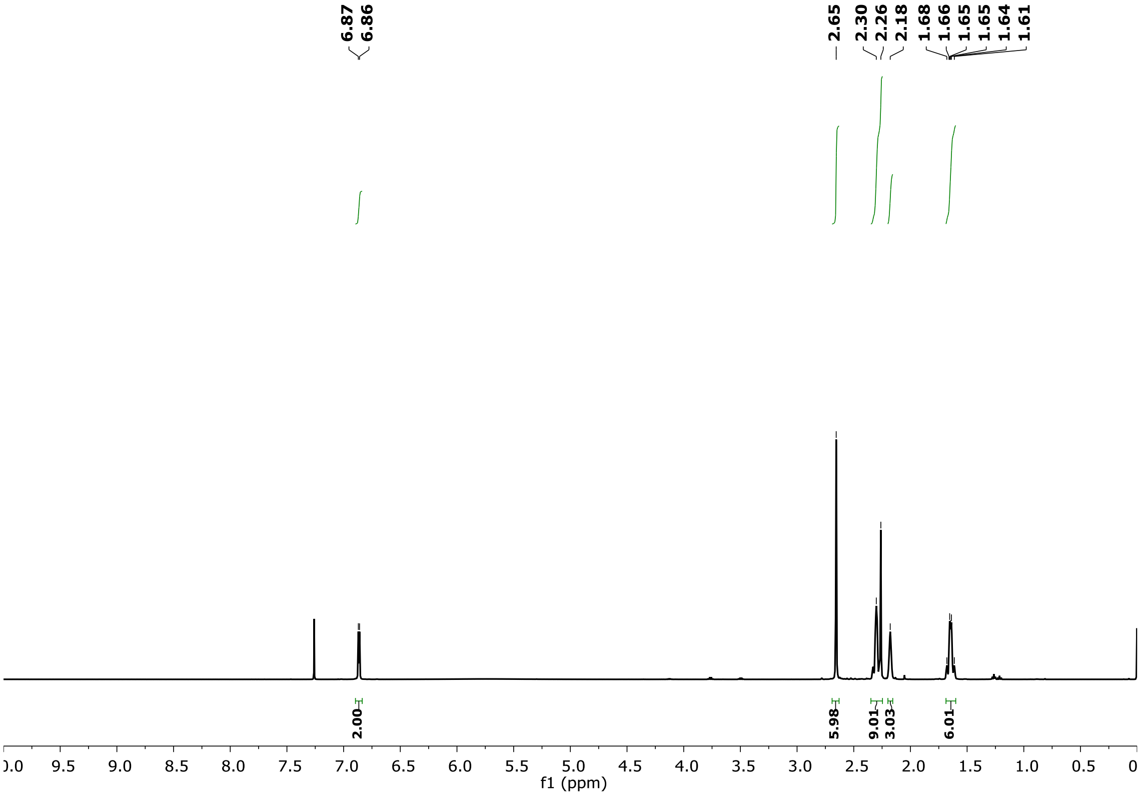

^{13}C NMR {^{1}H} (125.8 MHz, $CDCl_3$)

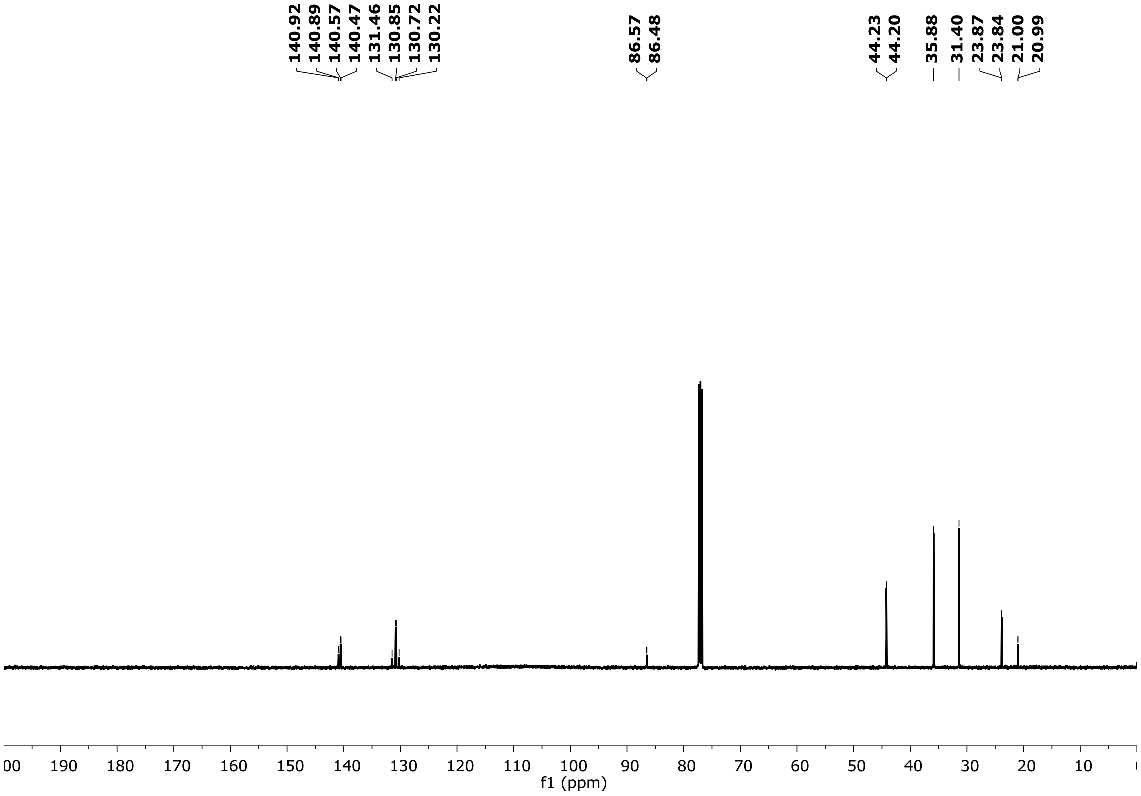

1-adamantyl 1-naphtylphosphonothioic acid (**2e**)

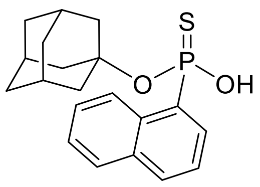

^{31}P NMR {^{1}H} (202.5 MHz, $CDCl_3$)

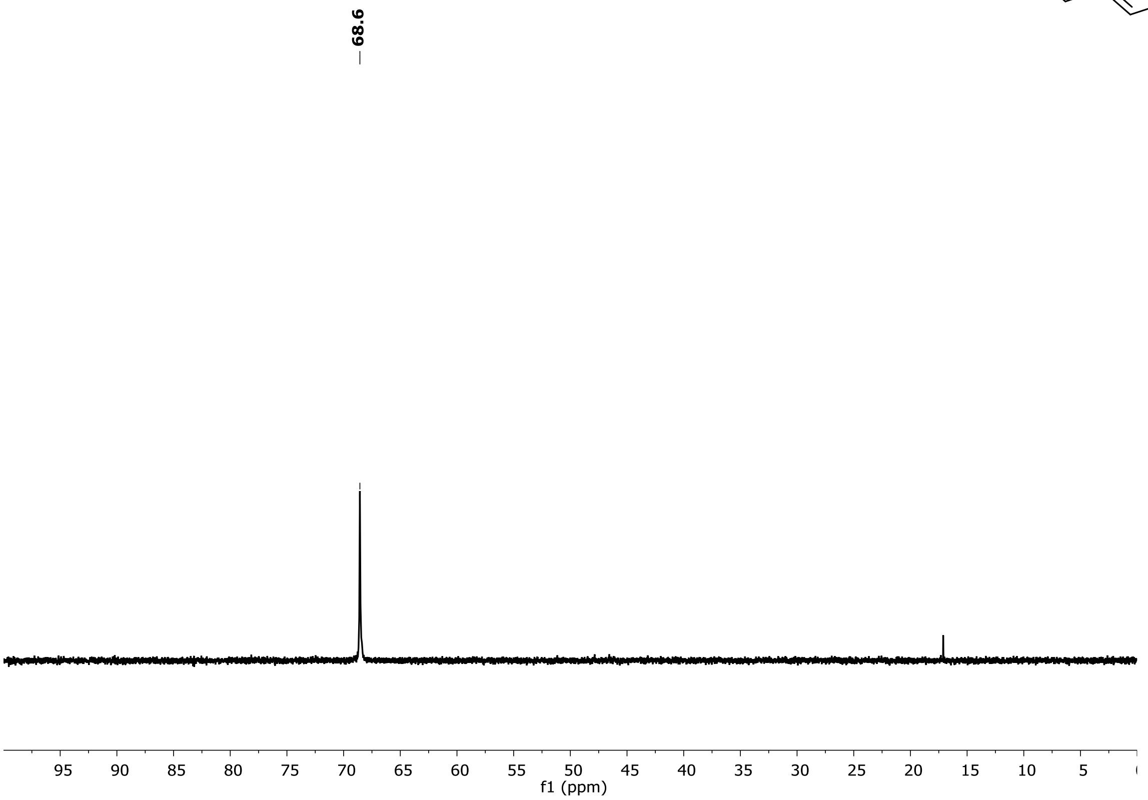

^{1}H NMR (500 MHz, $CDCl_3$)

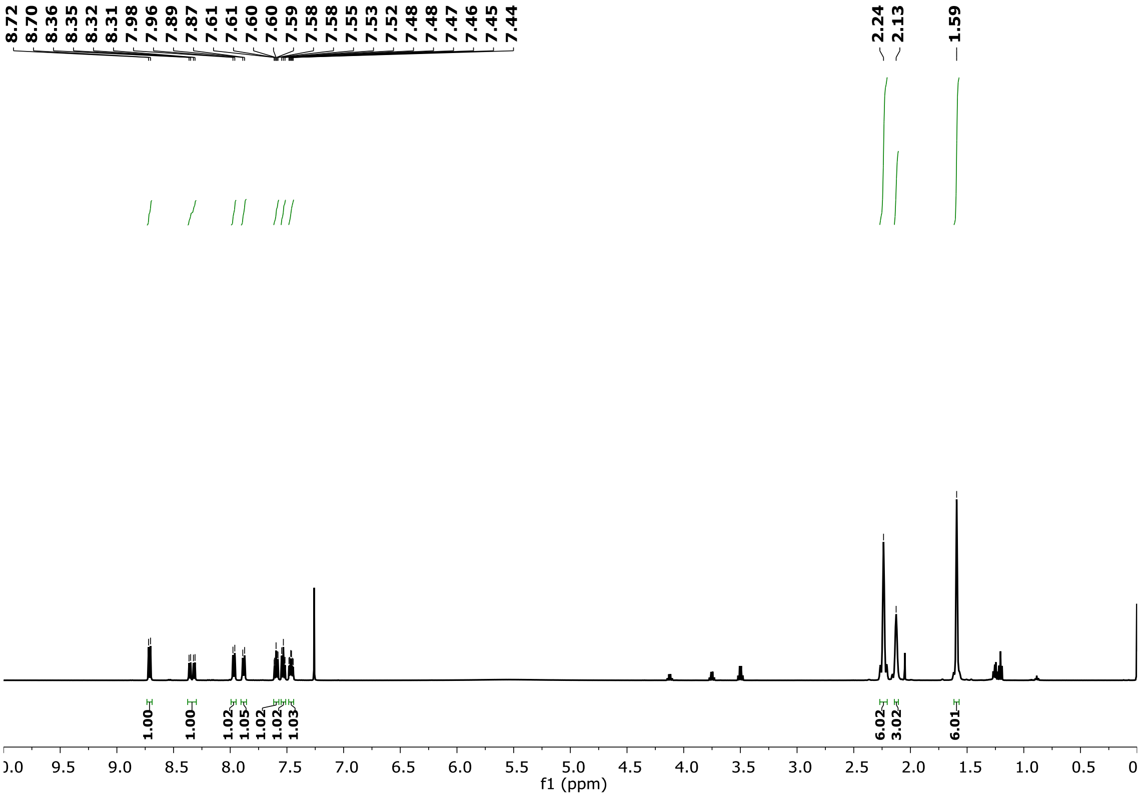

^{13}C NMR {^{1}H} (125.8 MHz, $CDCl_3$)

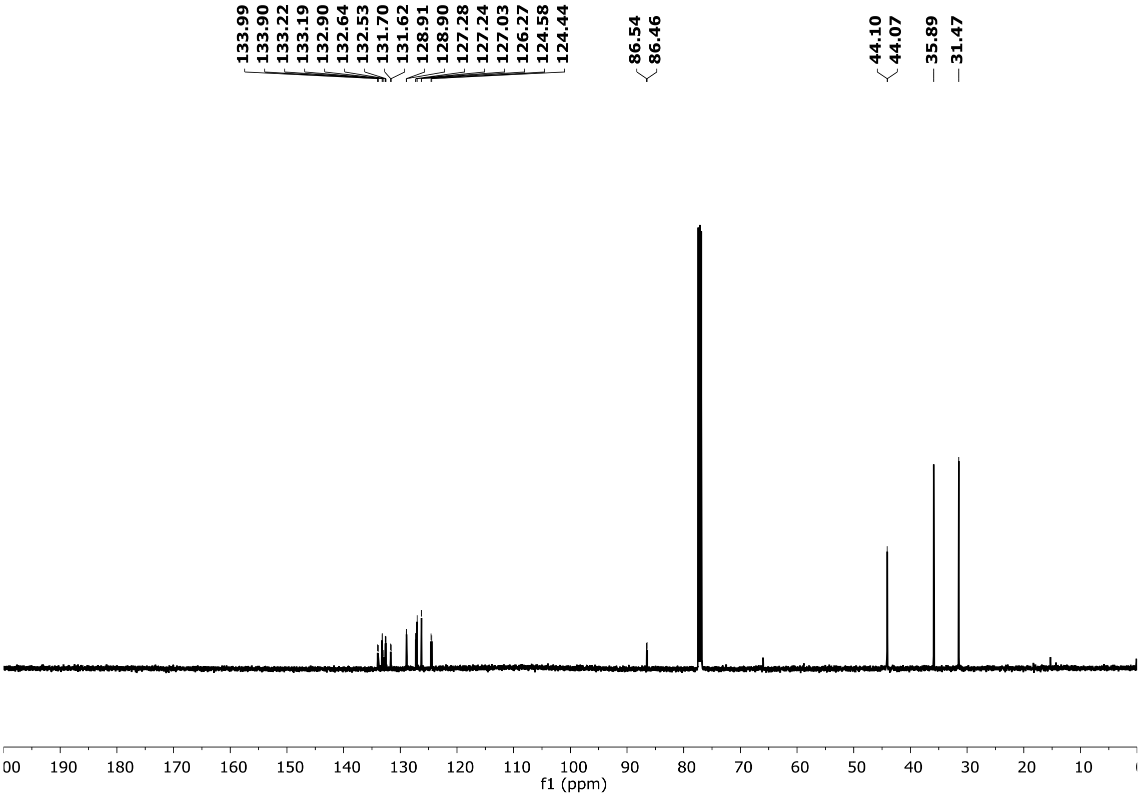

(*S*)-4-methoxy-*N*-(1-phenylethyl)aniline [(*S*)-**5**]

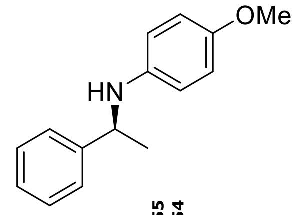

^{1}H NMR (500 MHz, $CDCl_3$)

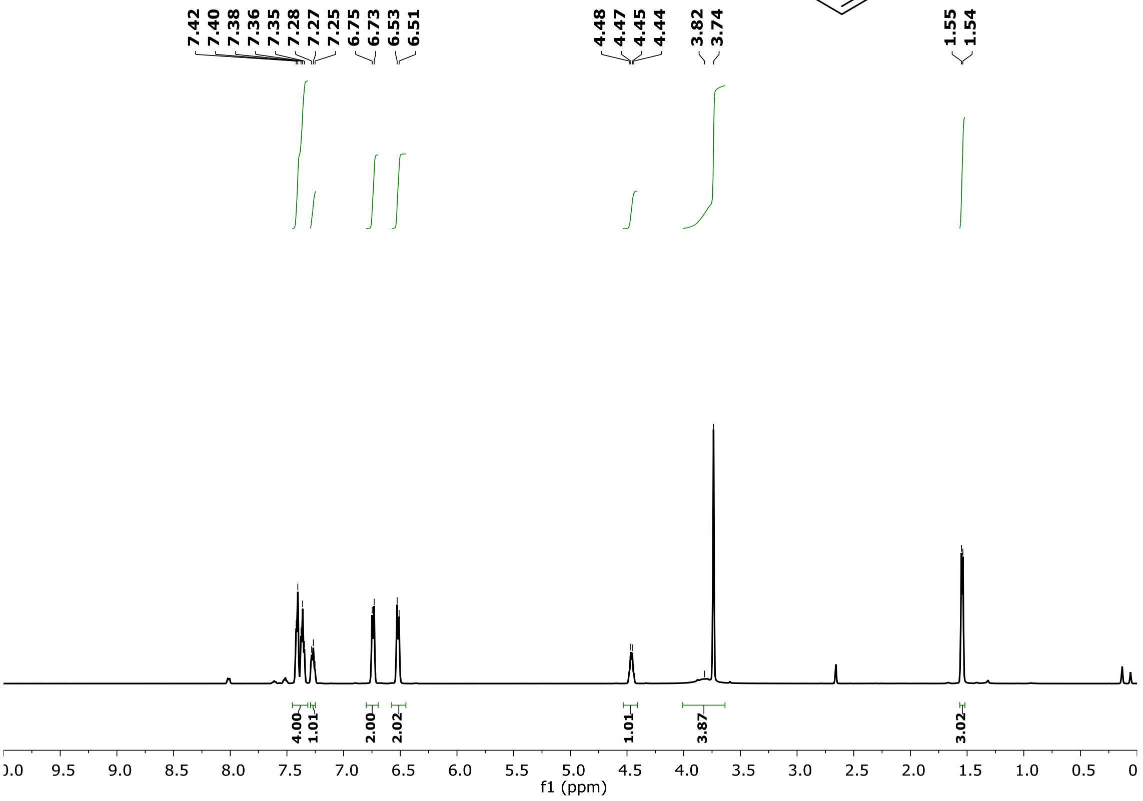

^{13}C{^{1}H} NMR (125.8 MHz, $CDCl_3$)

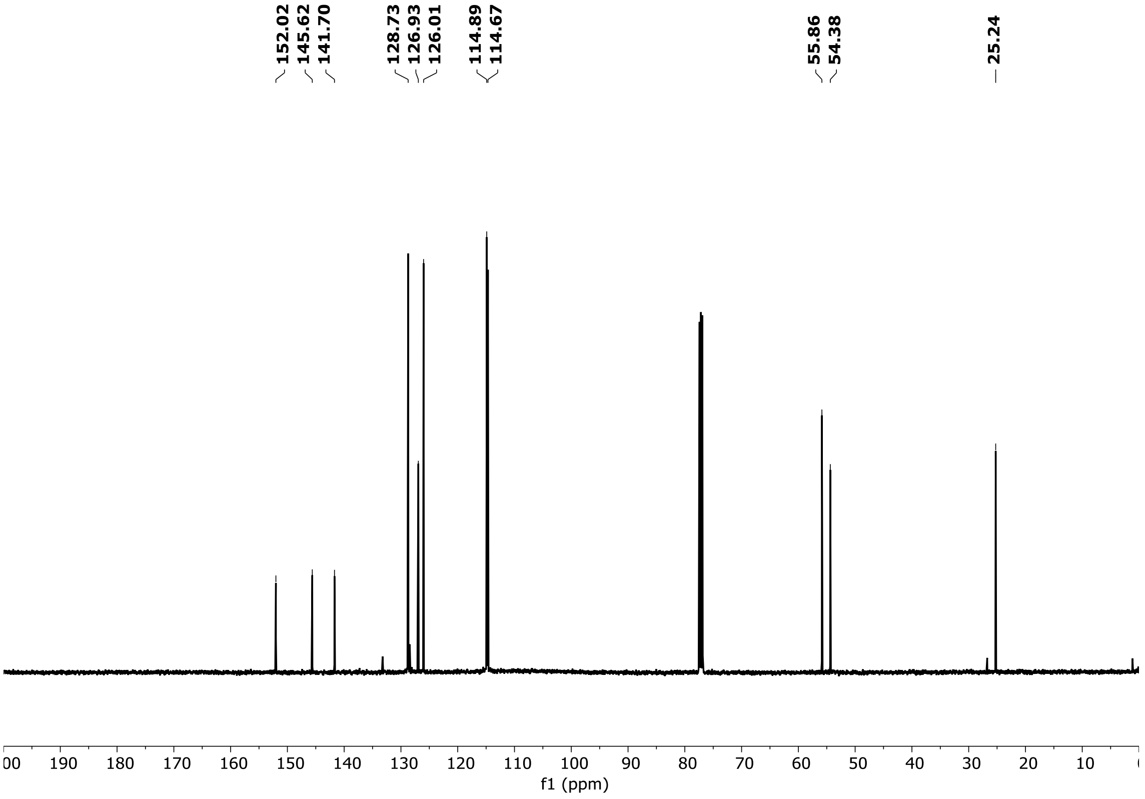

(*R*)-2-methyl-1,2,3,4-tetrahydroquinoline [(*R*)-**7**]

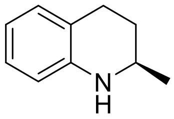

^{1}H NMR (500 MHz, $CDCl_3$)

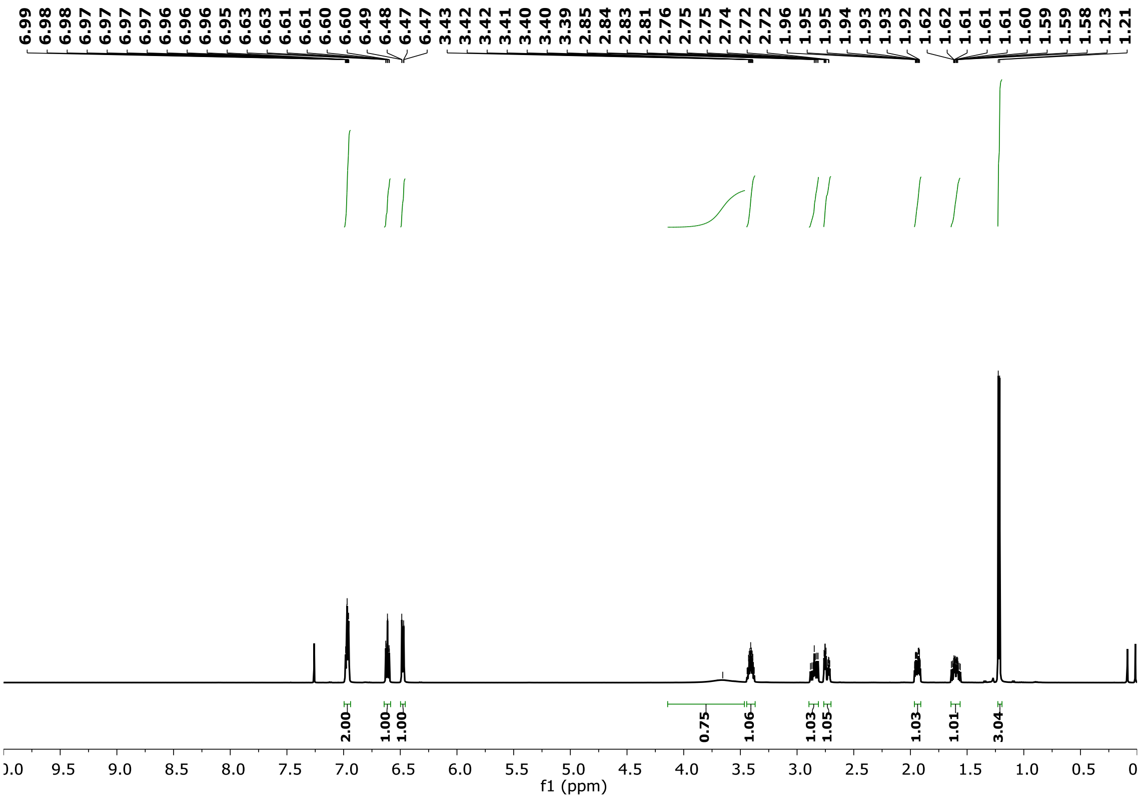

^{13}C{^{1}H} NMR (125.8 MHz, $CDCl_3$)

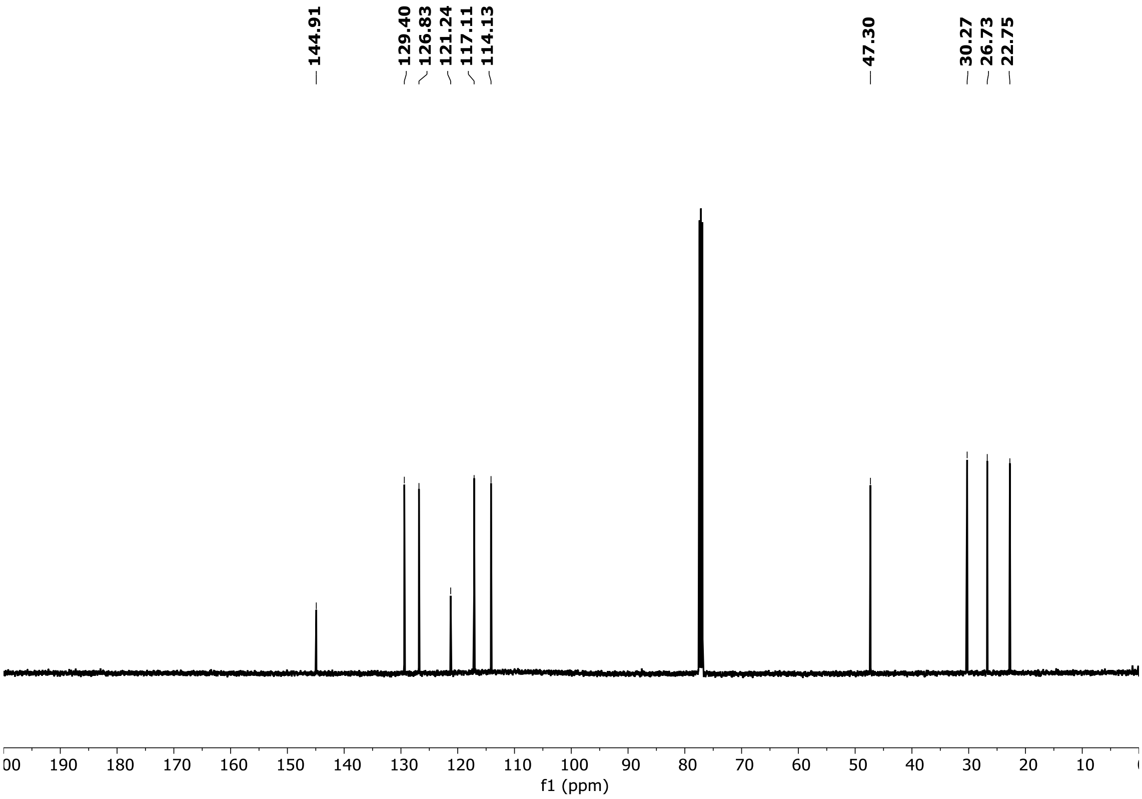

HPLC and NMR traces of optically active compounds (1-2)

1-adamantyl phenyl-*H*-phosphinate (**1a**)

Racemic

Peak #	Time [min]	Area [µV·s]	Height [µV]	Area [%]	Norm. Area [%]	BL	Area/Height [s]
1	7.548	4663239.90	391840.61	49.85	49.85	*BB	11.9009
2	13.801	4690666.03	168118.18	50.15	50.15	*BB	27.9010
		9353905.93	559958.78	100.00	100.00		

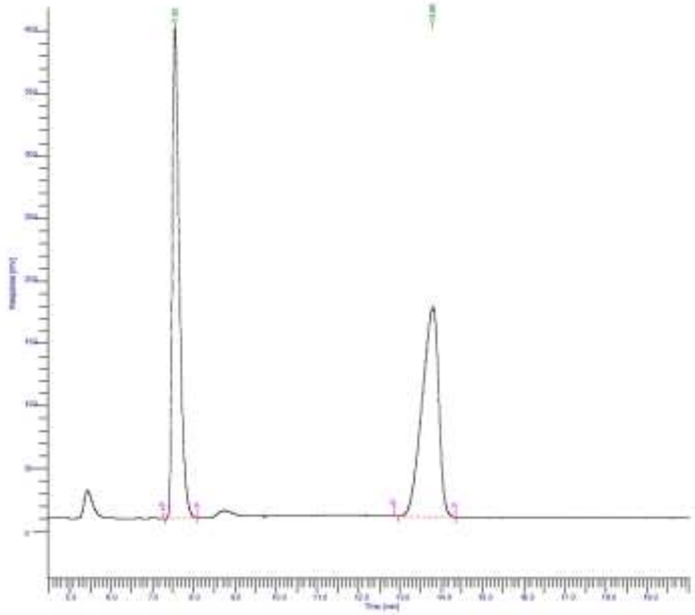

(*R*)-**1a**

Peak #	Time [min]	Area [µV·s]	Height [µV]	Area [%]	Norm. Area [%]	BL	Area/Height [s]
1	7.668	64565.86	5260.18	0.99	0.99	*BB	12.2745
2	13.974	6429577.46	207023.85	99.01	99.01	*BB	31.0572
		6494143.32	212284.03	100.00	100.00		

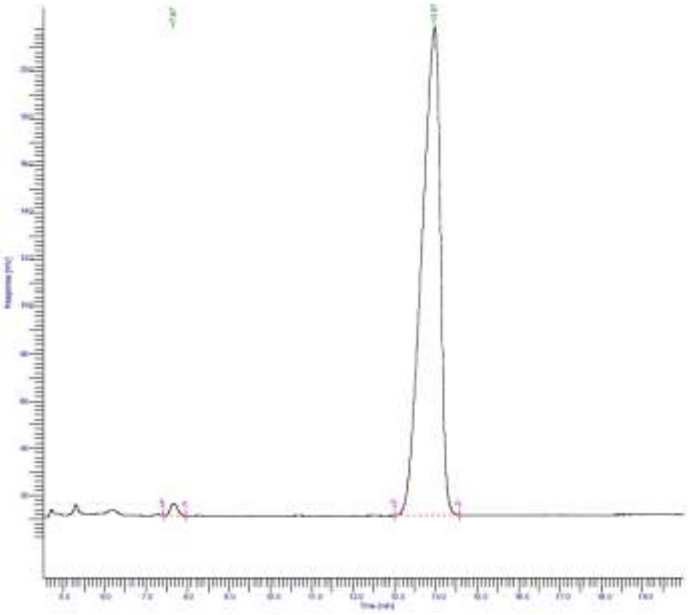

1-adamantyl (4-methoxyphenyl)-*H*-phosphinate (**1b**)

Racemic

Peak #	Time [min]	Area [µV·s]	Height [µV]	Area [%]	Norm. Area [%]	BL	Area/Height [s]
1	8.952	10323180.41	731715.44	47.74	47.74	*BB	14.1082
2	27.455	11301862.90	158468.88	52.26	52.26	*BB	71.3191
		21625043.31	890184.32	100.00	100.00		

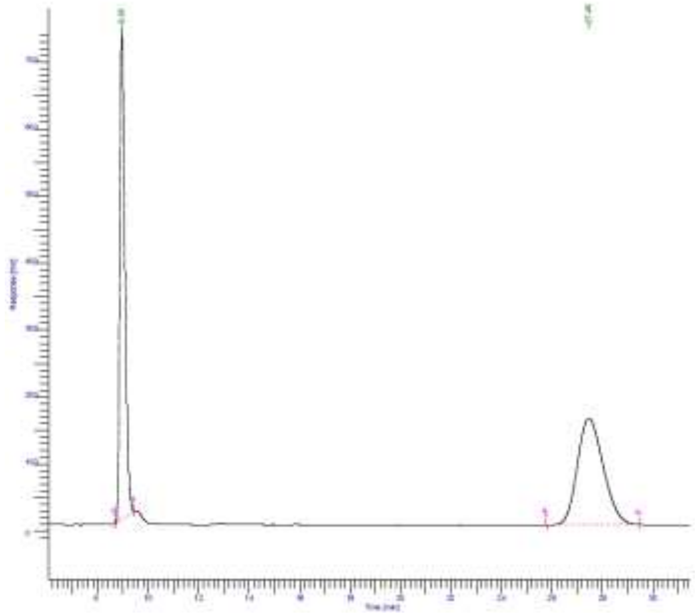

(*R*)-**1b**

Peak #	Time [min]	Area [µV·s]	Height [µV]	Area [%]	Norm. Area [%]	BL	Area/Height [s]
1	8.880	33181.99	2487.25	2.29	2.29	*BB	13.3408
2	27.048	1416905.12	20221.09	97.71	97.71	*BB	70.0707
		1450087.11	22708.33	100.00	100.00		

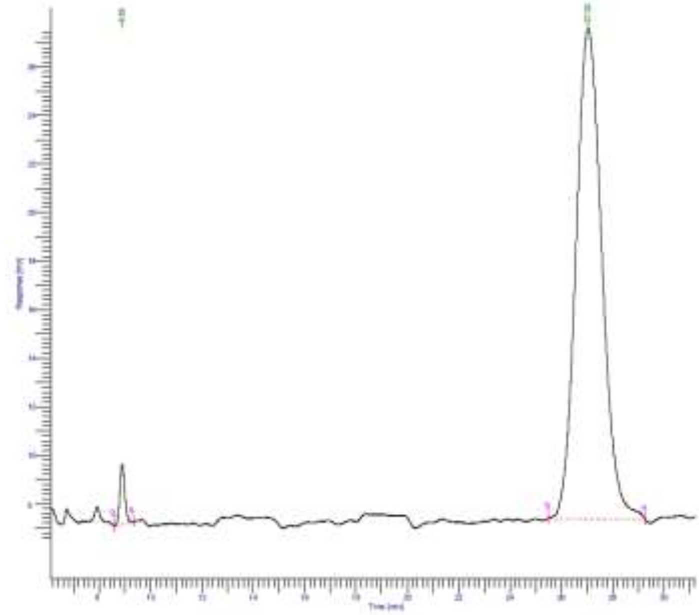

1-adamantyl (4-trifluoromethylphenyl)-*H*-phosphinate (**1c**)

Racemic

Peak #	Time [min]	Area [µV·s]	Height [µV]	Area [%]	Norm. Area [%]	BL	Area/Height [s]
1	6.029	3026755.78	335608.37	49.55	49.55	*BB	9.0187
2	7.699	3081510.13	195541.19	50.45	50.45	*BB	15.7589
		6108265.90	531149.57	100.00	100.00		

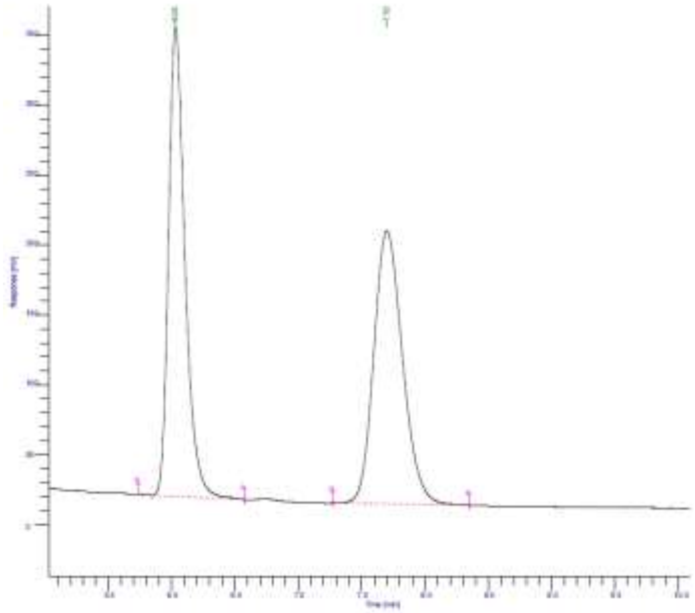

(*R*)-**1c**

Peak #	Time [min]	Area [µV·s]	Height [µV]	Area [%]	Norm. Area [%]	BL	Area/Height [s]
1	5.955	37198.50	4350.41	1.83	1.83	*BB	8.5506
2	7.585	1995727.08	128803.76	98.17	98.17	*BB	15.4943
		2032925.57	133154.17	100.00	100.00		

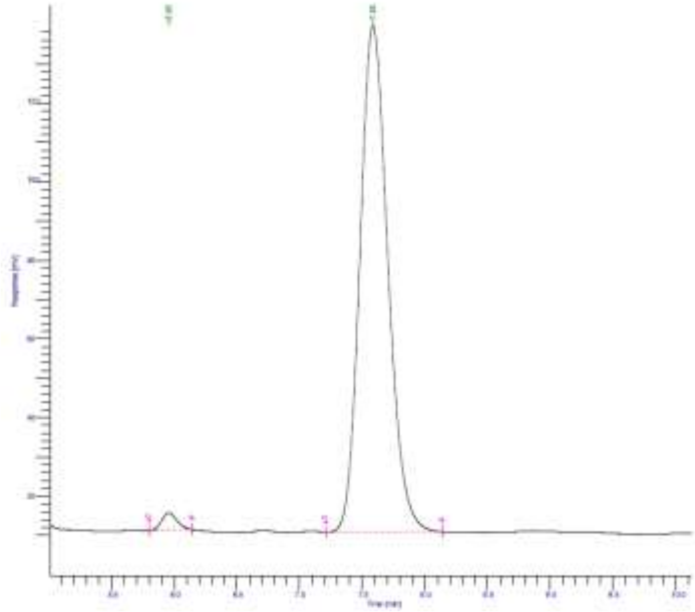

4-methoxy-N-(1-phenylethyl)aniline (**5**)

Racemic

Peak #	Time [min]	Area [µV·s]	Height [µV]	Area [%]	Norm. Area [%]	BL	Area/Height [s]
1	9.180	10606677.71	974861.97	49.66	49.66	BV	10.8802
2	9.659	10751191.08	928636.04	50.34	50.34	*VB	11.5774
		21357868.79	1.90e+06	100.00	100.00		

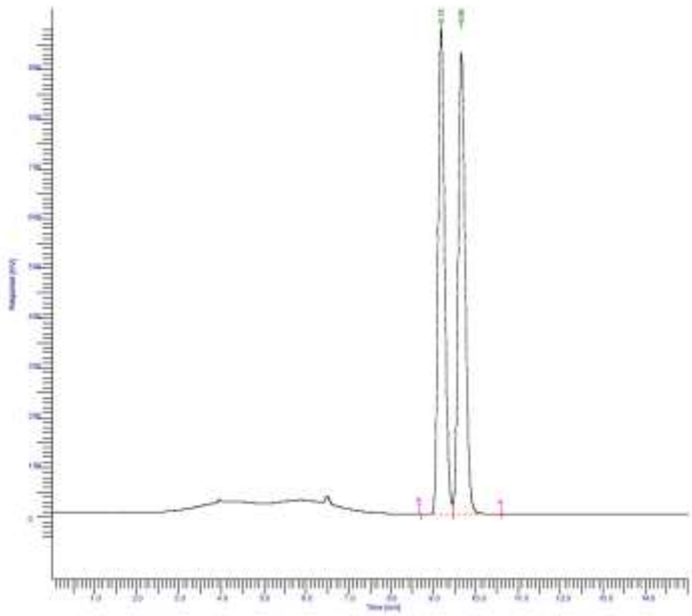

(*S*)-**5**

Peak #	Time [min]	Area [µV·s]	Height [µV]	Area [%]	Norm. Area [%]	BL	Area/Height [s]
1	9.996	9340453.42	758892.92	55.12	55.12	BV	12.3080
2	10.589	7604119.85	584064.33	44.88	44.88	*VB	13.0193
		16944573.27	1.34e+06	100.00	100.00		

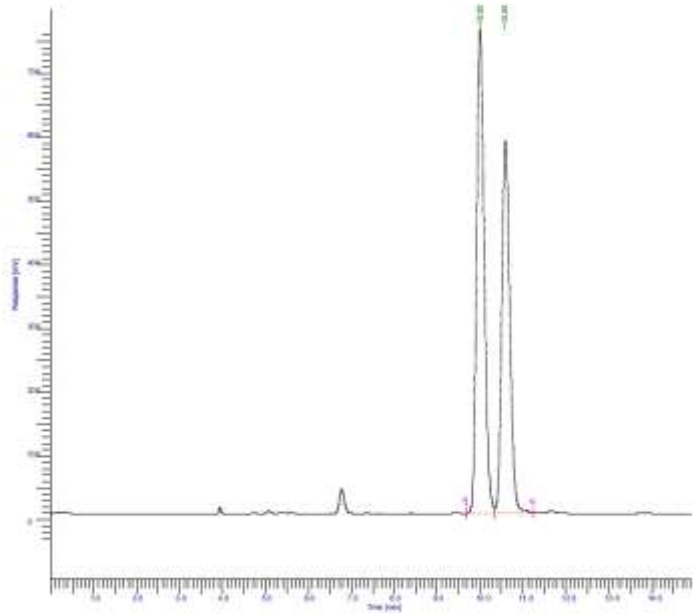

2-methyl-1,2,3,4-tetrahydroquinoline (**7**)

Racemic

Name	Retention Time	Area	Area Percent	Integration Codes
	9.333	8736752	49.803	Mx
	10.728	8806044	50.197	xM
Totals		17542796	100.000	

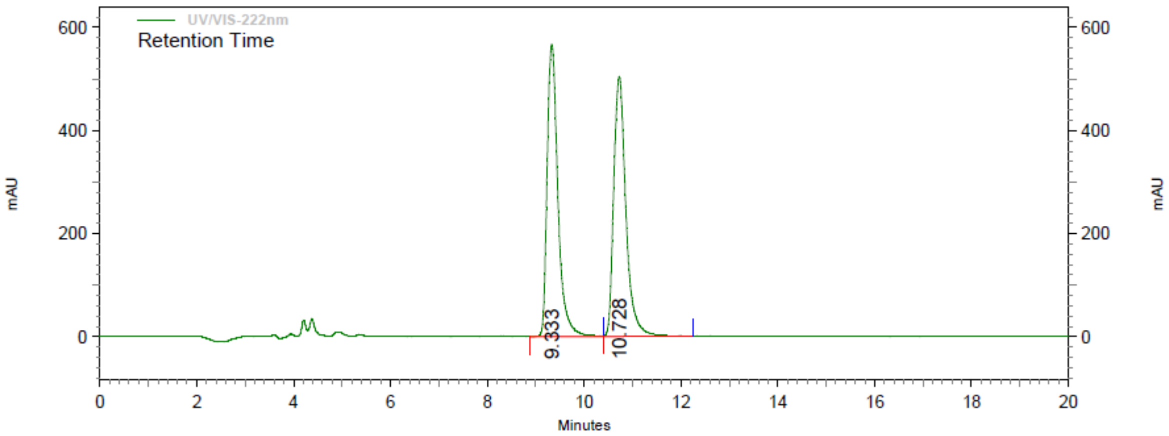

(*R*)-**7**

Name	Retention Time	Area	Area Percent	Integration Codes
	9.358	5839095	46.816	Mx
	10.745	6633419	53.184	xM
Totals		12472514	100.000	

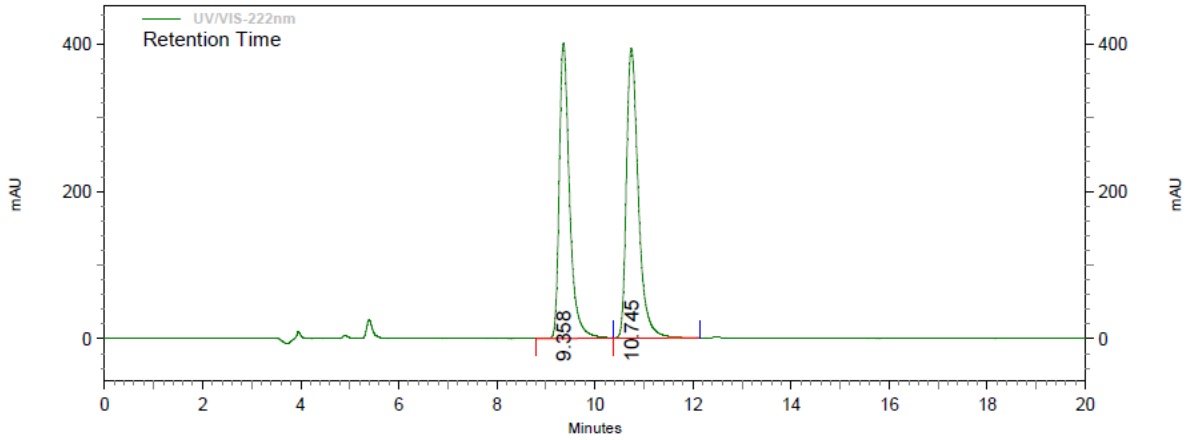

1-adamantyl phenylphosphonothioic acid (**2a**)

The enantiomeric excess (*ee*) values of **2a** were determined by ^{31}P NMR using 6.2 mg (20 μmol) of the analyte, 3.9 μL (30 μmol) (*S*)-phenylethylamine (**3a**) as CSA and 750 μL $CDCl_3$ as solvent.

Racemic **2a** + (*S*)-**3a**

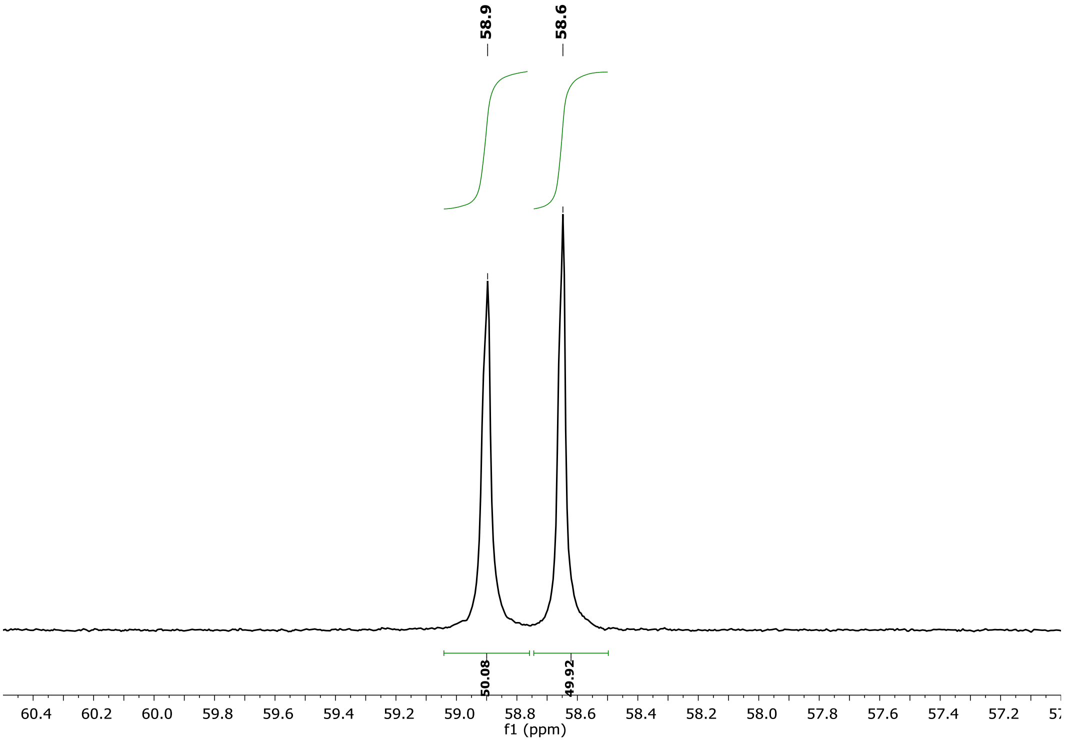

(*S*)-**2a** + (*S*)-**3a**

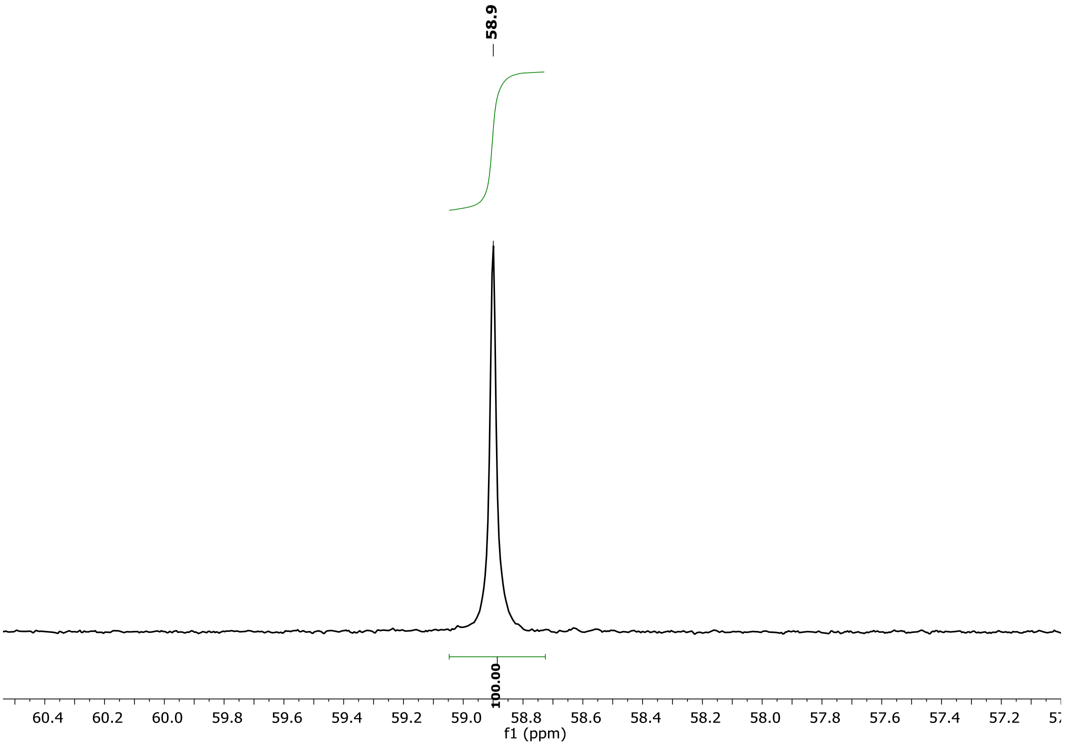

1-adamantyl (4-methoxyphenyl)phosphonothioic acid (**2b**)

The enantiomeric excess (*ee*) values of **2b** were determined by ^{31}P NMR using 6.8 mg (20 μmol) of the analyte, 3.9 μL (30 μmol) (*S*)-phenylethylamine (**3a**) as CSA and 750 μL $CDCl_3$ as solvent.

Racemic **2b** + (*S*)-**3a**

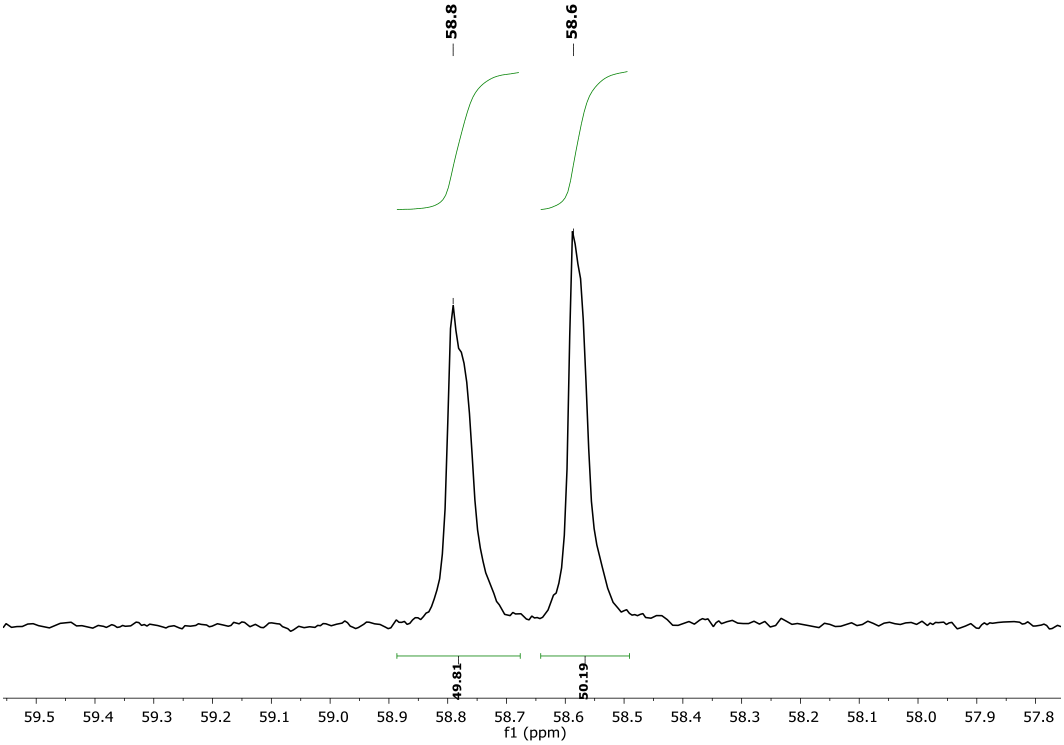

(*S*)-**2b** + (*S*)-**3a**

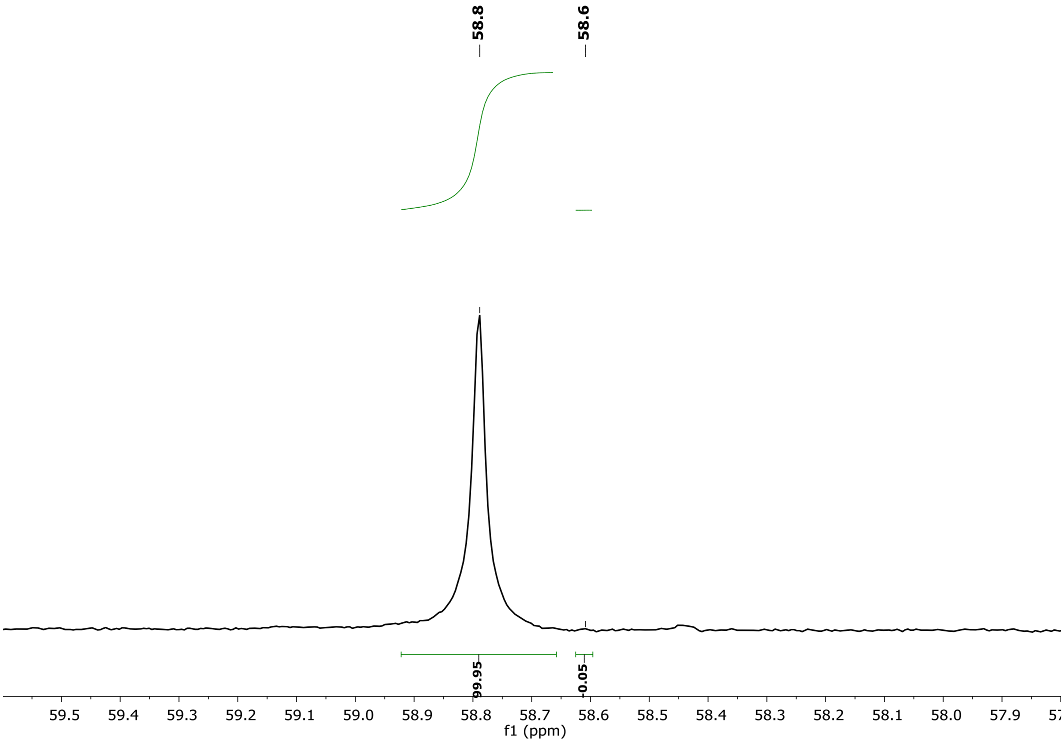

1-adamantyl (4-trifluoromethylphenyl)phosphonothioic acid (**2c**)

The enantiomeric excess (*ee*) values of **2c** were determined by ^{31}P NMR using 7.5 mg (20 μmol) of the analyte, 3.9 μL (30 μmol) (*S*)-phenylethylamine (**3a**) as CSA and 750 μL $CDCl_3$ as solvent.

Racemic **2c** + (*S*)-**3a**

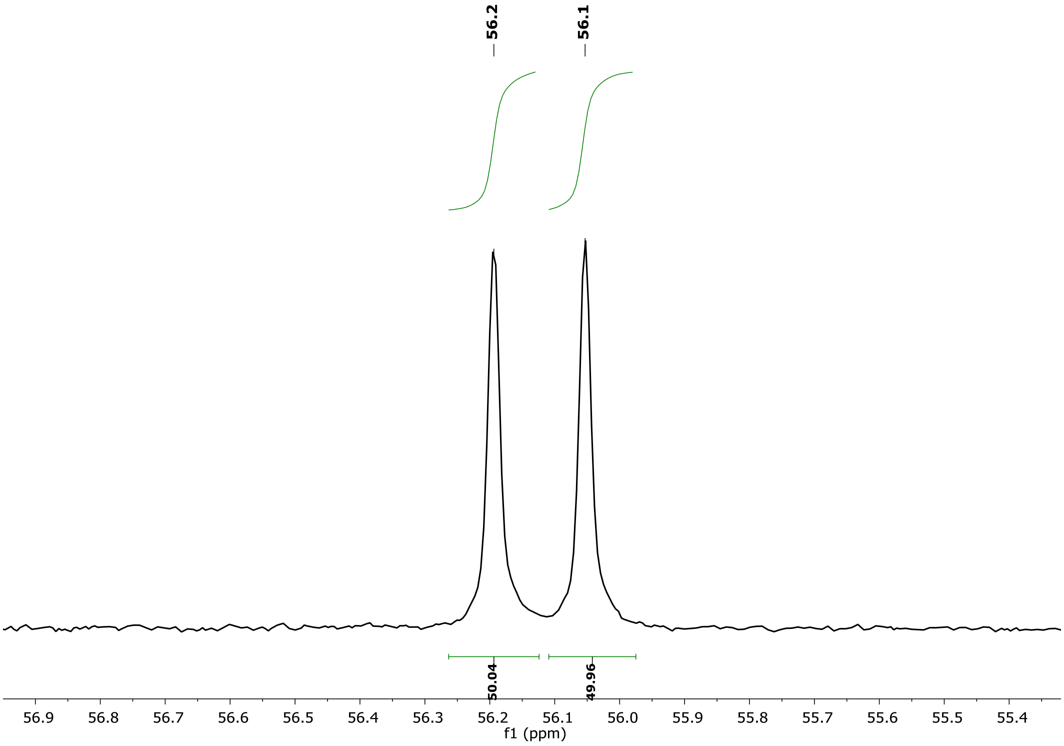

(*S*)-**2c** + (*S*)-**3a**

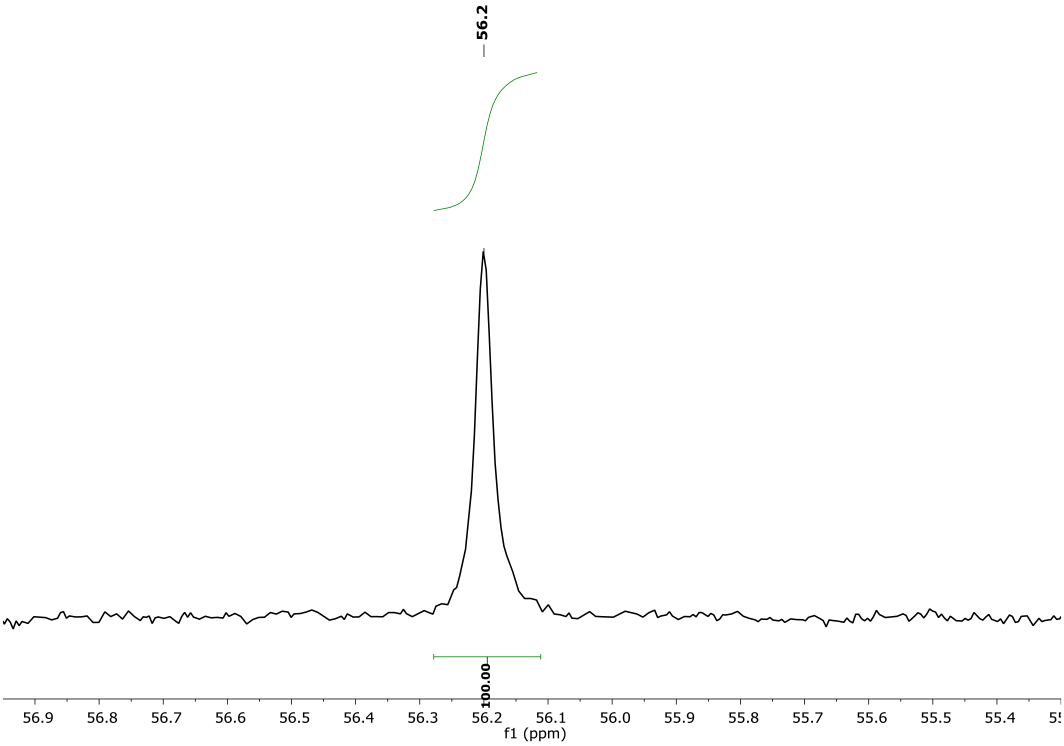

1-adamantyl mesitylphosphonothioic acid (**2d**)

The enantiomeric excess (*ee*) values of **2d** were determined by ^{31}P NMR using 7.0 mg (20 μmol) of the analyte, 3.9 μL (30 μmol) (*S*)-phenylethylamine (**3a**) as CSA and 750 μL $CDCl_3$ as solvent.

Racemic **2d** + (*S*)-**3a**

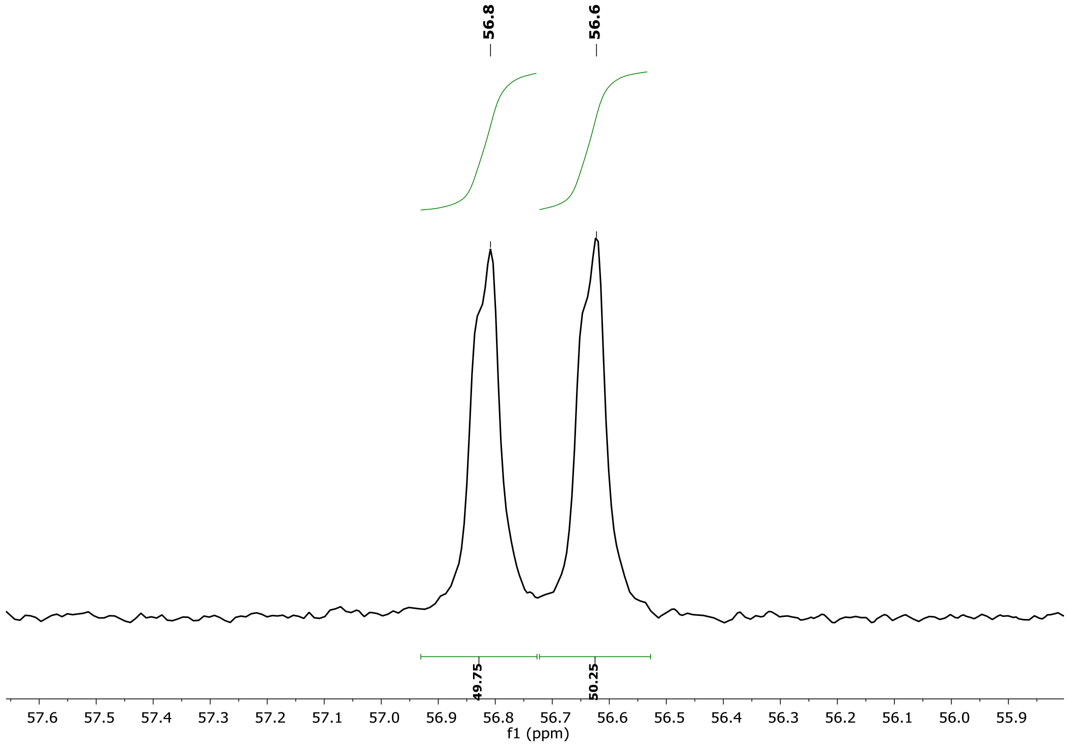

1-adamantyl 1-napthylphosphonothioic acid (**2e**)

The enantiomeric excess (*ee*) values of **2e** were determined by ^{31}P NMR using 7.2 mg (20 μmol) of the analyte, 3.9 μL (30 μmol) (*S*)-phenylethylamine (**3a**) as CSA and 750 μL $CDCl_3$ as solvent.

Racemic **2e** + (*S*)-**3a**

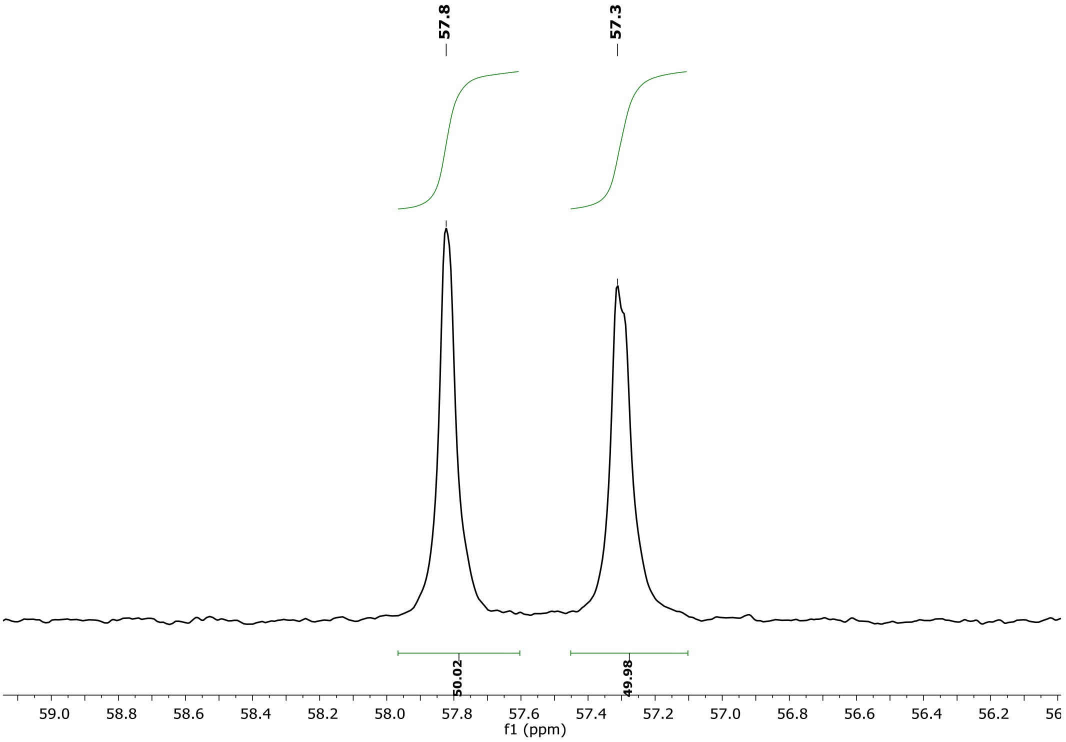

References:

1. Sheldon, R.A. *Chirotechnology: Industrial Synthesis of Optically Active Compounds*; Marcel Dekker: New York, 1993;
2. Han, J.; Wzorek, A.; Klika, K.D.; Soloshonok, V.A. Recommended Tests for the Self-Disproportionation of Enantiomers (SDE) to Ensure Accurate Reporting of the Stereochemical Outcome of Enantioselective Reactions. *Molecules* **2021**, *26*, 2757, doi:10.3390/molecules26092757.
3. Varga, B.; Vincze, D.; Pető, H.; Buna, L.; Pauló, J.; Holczbauer, T.; Mátravölgyi, B.; Hegedűs, L.; Fogassy, E.; Keglevich, G.; et al. Resolution of Aryl- H -Phosphinates Applied in the Synthesis of P-Stereogenic Compounds Including a Brønsted Acid NMR Solvating Agent. *Org. Chem. Front.* **2022**, *9*, 2797–2807, doi:10.1039/D2QO00241H.
